# Dietary mycotoxin exposure and human health risks: A protocol for a systematic review

**DOI:** 10.1016/j.envint.2024.108456

**Published:** 2024-02

**Authors:** T. Goessens, T. Mouchtaris-Michailidis, K. Tesfamariam, N.N. Truong, F. Vertriest, Y. Bader, S. De Saeger, C. Lachat, M. De Boevre

**Affiliations:** a Center of Excellence in Mycotoxicology and Public Health, Faculty of Pharmaceutical Sciences, Ghent University, Ghent, Belgium; b Department of Food Technology, Safety and Health, Faculty of Bioscience Engineering, Ghent University, Ghent, Belgium; c Ghent University, Department of Internal Medicine and Pediatrics, Faculty of Medicine and Health Sciences, Ghent, Belgium

## Abstract

**Background:**

Mycotoxins are toxic fungal secondary metabolites that contaminate a wide spectrum of essential foods worldwide, such as grain-based products, nuts and spices, causing adverse health effects pertaining to their carcinogenic, nephrotoxic and hepatotoxic nature, among others.

**Aim:**

The aim of this systematic review (SR) is to systematically search for, appraise and synthesize primary research evidence to identify what is known about dietary mycotoxin-related health effects and what remains unknown, as well as the uncertainty around findings and the recommendations for the future.

**Search strategy and eligibility criteria:**

Search strategies, as well as eligibility criteria were structured according to a predefined PECO (population, exposure, comparison, and outcome) research question and developed in an iterative scoping process. Several bibliographic databases, including Embase, Cochrane Library, Pubmed, Web of Science Core Collection and Scopus, will be searched. Primary research on any measured or modelled dietary exposure to a single or multiple mycotoxins, and adverse human health outcomes (*i.e.* cancer, non-carcinogenic diseases, and reproductive & developmental adverse outcomes) will be included, and references will be imported into Covidence. *In vitro*, *ex vivo*, *in silico*, animal and review studies, as well as expert’s opinions, secondary literature, conference abstracts, presentations, posters, book chapters, dissertations and studies involving non-dietary mycotoxin exposure, will be excluded.

**Study selection:**

Two independent reviewers will screen titles and abstracts, and review full-texts. Any disagreements will be resolved by a third reviewer based on two-third majority.

**Data extraction:**

Data from retained eligible studies will be extracted by the principal reviewer, and peer-checked by a second reviewer.

**Study quality assessment:**

Eligible studies will be evaluated for risk of bias (Overall High-Quality Assessment Tool, OHAT) and certainty of evidence (Grading of Recommendations Assessment, Development and Evaluation, GRADE).

**Evidence synthesis:**

A detailed summary of the included studies will be provided within a tabular format and narratively discussed. Heat maps will be constructed to provide information on available knowledge (gaps), and a *meta*-analysis may be performed based on the variability in predefined PECO elements and depending on the heterogeneity of studies.

**Conclusion:**

This protocol describes the methodology for the conduct of a SR on mycotoxin-related human health risks, that could guide future research and inform regulatory decisions, as emphasized by the European Commission within the field of regulatory risk assessment for emerging chemicals.

## 1 Introduction

Mycotoxins are toxic fungal secondary metabolites that contaminate a wide spectrum of essential foods worldwide, including staple crops consumed by the most vulnerable populations. Although many filamentous moulds are toxigenic, the most important mycotoxin-producing genera are *Aspergillus*, *Fusarium* and *Penicillium* (Pitt and David Miller, 2017). Because one fungal species may produce different mycotoxins, and the same mycotoxin may also be produced by several species, there is a very high likelihood of multiple mycotoxins co-occurring in food and feed products (De Ruyck et al., 2015). Eskola et al. (2020) estimated that between 60 and 80 % of the world’s food crops are contaminated by mycotoxins (Eskola et al., 2020). Therefore, human exposure to one or more mycotoxins is extensive. Previous studies have demonstrated mycotoxin exposure to be highly prominent in Belgium and Europe (De Ruyck et al., 2020, Heyndrickx et al., 2015), and it has been predicted to become an even greater food safety issue considering a + 2 °C global average temperature scenario as part of climate change for the upcoming years (Battilani et al., 2016, Mitchell et al., 2016, Wilson and Dahl, 2018, Wu, 2013, Wu and Mitchell, 2016). Unfortunately, low- and middle-income countries are even more susceptible to the harmful effects of mycotoxins due to their diet which is mainly based on susceptible staple crops. In addition, regulations are less stringent or not implemented resulting in significantly higher contamination levels (De Ruyck et al., 2015). Financially, mycotoxin contamination has resulted in global economic losses due to increased need for testing, loss of loads/lower prices and decreased production in livestock industry (Mitchell et al., 2016, Wilson and Dahl, 2018).

Based on their diverse physicochemical properties, mycotoxins contribute to a diversity of adverse human health outcomes (Riley et al., 2018). The foremost toxic effects pertain to their carcinogenicity, nephrotoxicity, hepatotoxicity, estrogenicity, neurotoxicity and reproductive and immune system alterations. The most common and pathologically-significant mycotoxins are aflatoxins (*e.g.* aflatoxin B1, AFB1), fumonisins (*e.g.* fumonisin B1, FB1), ochratoxins (*e.g.* ochratoxin A, OTA), trichothecenes (*e.g.* deoxynivalenol, DON), patulin (PAT) and zearalenone (ZEN) (Peraica et al., 1999a, Truckness, 1994, Ueno and Hsieh, 1985). Aflatoxins specifically target the liver, causing liver toxicity through mechanisms such as interference with normal protein synthesis, oxidative stress, and damaging liver cells. Fumonisins have been associated with a range of adverse health outcomes in children, including neural tube defects, embryonic and fetal toxicity as well as growth impairments in children (Pierron et al., 2016b), whilst trichothecenes mainly affect the rapid proliferating tissues such as the hematopoietic, lymphoid and gastrointestinal tissues leading to abdominal pain, vomiting, diarrhoea and growth retardation (Mahato et al., 2022). OTA is known as a nephrotoxin, with the kidney being the primary target organ, associated with human Balkan endemic nephropathy (BEN), renal failure and renal cancer (Kumar et al., 2020). PAT has been associated with gastrointestinal problems (Mahato et al., 2021b) whilst ZEN causes reproductive disorders and an advanced puberty time (Mahato et al., 2021a). Furthermore, mycotoxins such as DON have been demonstrated to cause alterations in gut microbiota (Saint-Cyr et al., 2013).

Although various mycotoxin-related literature reviews have been conducted, either focusing on a single mycotoxin or most common health effects, a comprehensive review that covers all existing scientific information relevant to the overarching burden of mycotoxins on human health, and addressing co-exposure risks, in a systematic manner is lacking (De Ruyck et al., 2015, Kyei et al., 2020, Mahato et al., 2021a). Among existing evidence synthesis methods, a systematic review (SR) is a review of evidence with a clearly formulated question that uses systematic and explicit methods to identify, select, and critically appraise relevant primary research, and to extract and analyze data from the studies that are included in the review. The purpose of the SR is to obtain a complete summary of existing primary research in response to a research question, and to identify potential knowledge gaps. Traditional literature reviews often lack thoroughness and precision and are conducted *ad hoc*, rather than following a specific methodology, raising questions about the quality and trustworthiness of the data (Snyder, 2019). This manuscript outlines a protocol for a SR of human health risks related to dietary mycotoxin exposure to support evidence-based decision-making and policy-making.

## 2 Objectives

The overall objective of this SR is to systematically search for, appraise and synthesize primary research evidence to identify what is known about dietary mycotoxin-related health effects and what remains unknown, as well as the uncertainty around findings and the recommendations for the future. While individual mycotoxins have been extensively studied, there is a need for more comprehensive SRs that can help to identify patterns and associations between exposure to individual as well as multiple mycotoxins, and adverse health outcomes (De Ruyck et al., 2015, Kyei et al., 2020, Mahato et al., 2021a). The work follows up on the call for SRs by the European Commission in the field of regulatory risk assessment of emerging chemicals (European Commission - Horizon Europe Programme, 2021).

Specific objectives are the following:

• Identify and appraise available literature on the association between any type of measured or modelled, single or multiple, dietary mycotoxin exposure for 69 mycotoxins listed in Table 1, and adverse health outcomes, in humans of all age, gender, health status and life stage.

**Table 1:** Critical research questions as building blocks for framing the PECO questions and data extraction template.

**Human health risk**
Health effects: there is a growing concern about emerging or less-studied mycotoxins, *e.g.* beauvericin, enniatins and *Alternaria* mycotoxins. Research is needed to understand the occurrence, toxicological properties, and health effects of these newly identified mycotoxins (Gruber-Dorninger et al., 2017).
*Q = What are the human health effects associated with dietary exposure to mycotoxins, and by extent emerging mycotoxins?*
**Exposure**
Analyzed matrix: accurate assessment of mycotoxin exposure is crucial for understanding the health risks associated with these compounds. In contrast to exposure assessment via biomarker analysis, assessment through consumption and food concentration data relies on self-reported data which may be subject to recall bias or reporting errors, and are limited by the availability and accuracy of the data for different food products (Vandenberg et al., 2023).
*Q = Has the dietary mycotoxin exposure been measured externally (via consumption and food concentration data) or internally (via biomarkers) in health risk association studies? Does this have an influence on the results?*
Multiple mycotoxins: in real-life, individuals are often exposed to multiple mycotoxins simultaneously. However, the majority of research has focused on individual mycotoxins rather than their combined effects. Further investigation is necessary to understand mycotoxin’s potential additive, synergistic, or antagonistic effects (Braun et al., 2020).
*Q = Have the established health risk association studies taken into account multiple mycotoxin-exposure?*
**Study design**
Study design: Several types of study have been utilized to investigate mycotoxin-associated health effects, *e.g.* cohort, cross-sectional and case-control studies. Often, the conduct of clinical trials remains limited, since it involves exposing individuals to different levels of mycotoxins below the threshold of toxicological concern, implying the need for highly sensitive detecting techniques and raising ethical concerns when pertaining to mycotoxins having carcinogenic potential (Vidal et al., 2018).
*Q = Does the type of study design influence the outcome of mycotoxin-associated health studies?*
**Regulation**
Regulatory frameworks: the establishment of regulatory frameworks is crucial to ensure the safety of food regarding mycotoxin contamination. Research is needed to support the development of evidence-based Maximum Residue Levels (MRLs) for mycotoxins in different food commodities. No MRLs are yet established for emerging mycotoxins. Other mycotoxins such as patulin are regulated in different food products in certain areas, but lack an universally established MRLs (European Commission, 2006).
*Q = which concentration of mycotoxin in food presents a substantial human health risk and does this concur with current regulation? Are there mycotoxins presenting a substantial human health risk for which no legislation has been yet obtained?*
• Identify areas of uncertainty and knowledge gaps concerning studied health outcomes per dietary mycotoxin.
• Make recommendations for future research and risk assessment activities, including population-based cohort studies and intervention trials, to support evidence-based decision- and policy-making.

## 3 Problem formulation

### 3.1 Scoping exercise

As proposed by (Schreier et al., 2022), a scoping exercise was performed to assess preliminary evidence synthesis and determine the relevance for a de novo systematic review. **First**, a series of critical research questions were constructed as building blocks for framing the PECO questions and data extraction template, addressing the knowledge gaps and research/regulatory needs of dietary mycotoxin-related health impacts (Table 1). **Second**, to guide the selection of exposure variables of the scoping review, a list of 69 major food- and beverage-related mycotoxins was constructed based upon literature (Chrevatidis, 2003, EFSA, 2017, EFSA, 2014, EFSA, 2012a, Frisvad et al., 2018, Han et al., 2014, Ismaiel and Papenbrock, 2015, Kagot et al., 2022, Knutsen et al., 2018, Krska and Crews, 2008, Mahato et al., 2021b, Martins et al., 2017, Mizutani et al., 2009, Nicholson, 2004, Ostry et al., 2013, Otero et al., 2020, Patriarca, 2016, Pierron et al., 2016a, Ropejko and Twarużek, 2021, Schamann et al., 2022, Schrenk et al., 2020, Sliwi, M.P., 2021, Streit et al., 2013, Zorzete et al., 2013) and expert consultation processes (TG, TMM, FV, YB, SDS and MDB), the latter resulting in the inclusion of secondary mycotoxin metabolites, *i.e.* the plant-produced 3- and 15-acetyldeoxynivalenol, as well as the microbiota-produced deepoxy-deoxynivalenol (Table 2). **Third,** as per MedDRA System Organ Class (Medical Dictionary for Regulatory Activities, 2022) and using the keywords (*i.e.* mycotoxins) identified in the previous step, adverse health were obtained from a literature (Google Scholar, Pubmed and Embase), as well as topic-specific organization websites from the European Food Safety Authority (EFSA, 2020), World Health Organization (WHO, 2018) and International Agency of Cancer (IARC, 2023). A final list of 15 mycotoxin-health-related outcomes were selected: cancer, kidney disease, liver disease, immunosuppression, gastrointestinal disease (Bennett et al., 2003), inflammatory bowel syndrome (Rao Jala et al., 2018), alimentary toxic aleukia (Peraica et al., 1999b), endocrine system disease (Kowalska et al., 2016), metabolic syndrome (Islam, 2017), Parkinson’s disease, Alzheimer’s disease (Arce-l et al., 2021), reproductive toxicity (Kowalska et al., 2016), developmental problems (Tesfamariam et al., 2019), cardiovascular disease (Wang et al., 2021) and altered microbiota (Rao Jala et al., 2018). **Finally**, detailed electronic search strings were developed in Pubmed, Embase, Cochrane Library, Web of Science Core Collection and Scopus, using keywords from the predefined list of major food- and beverage-related mycotoxins (Table 2), in combination with “systematic review”, “*meta*-analysis”, “evidence map” and “systematic search”, and without applying any filters, resulting in the output of 300, 425 and 3 articles, respectively (Table S1, S2 and S3 of Supplementary Material). Additionally, existing or ongoing reviews on the topic of mycotoxins and human health were searched in databases commonly used for registrations (PROSPERO, https://www.crd.york.ac.uk/prospero/, (n = 44); Open Science Framework (OSF) Registry, https://osf.io/registries, (n = 14); and Zenodo, https://zenodo.org/, (n = 16)) (Table S4 of Supplementary Material). Of the retrieved articles, 22 were directly related to the present work, being dedicated to either a subpopulation (*i.e.* pregnant women and/or children), single mycotoxin exposure (*i.e.* aflatoxins, trichothecenes, ochratoxins or zearalenone) and/or a specific health outcome (*i.e.* adverse pregnancy outcomes, child health, liver cirrhosis, liver cancer, breast and cervical cancer, renal disease, Kashin-Beck disease, immunotoxicity or female reproduction) (Table S5 of Supplementary Material). Many mycotoxin-related adverse health outcomes, including inflammatory bowel disease, gastrointestinal cancer, neurological disorders and altered microbiota, have not been covered by existing literature reviews. Additionally, of the retrieved articles, only 2 were constructed based upon a well-articulated PECO statement and a transparent valid literature search, risk of bias assessment and certainty assessment, raising questions about the quality and reliability of the review results (Table S5). Finally, large differences existed in studied mycotoxin & health outcome, as well as study design and variables such as mycotoxin matrix, resulting in substantial heterogeneity of evidence between reviews, justifying the need for a new review with a harmonised approach. More specific, an effectiveness review was considered the most relevant approach to assess the strength of evidence on mycotoxins and health, with particular focus to summarise size and direction of the effect, with respect to exposure, health outcome, study design and matrix (Roberts et al., 2006).

**Table 2:** Mycotoxins commonly found in food and food products.

**Fungal species**	**Mycotoxins**	**Food products**
*Aspergillus* spp.	Aflatoxins: AFB1, AFB2, AFG1, AFG2, AFM1 Aspertoxin Aspergillic acid Sterigmatocystin Hydroxy-aflavinine Ditryptophenaline Flavacol Hydroxyneoaspergillic acid Leporin C Methylcitreo-isocoumarin Paspalinine Speradine A Versiconol	Aflatoxins, aspertoxin & aspergillic acid: Maize, rice, nuts, oilseeds, dried fruit and spices (Schamann et al., 2022, Schrenk et al., 2020). Sterigmatocystin: cereal products, pistachio nuts, pecan nuts & green coffee beans (Chrevatidis, 2003). Hydroxy-aflavinine: tea & dried fruits (Frisvad et al., 2018). Ditryptophenaline: peanuts (Martins et al., 2017). Flavacol: maize (Frisvad et al., 2018, Kagot et al., 2022). Hydroxyneoaspergillic acid, leporin C, methylcitreo-isocoumarin, paspalinine, speradine A & versiconol: maize (Kagot et al., 2022).
*Fusarium* spp.	Trichothecenes: T2, HT2, NIV, DON, 15-ADON, 3-ADON, fusarenon X, neosolaniol, NX toxin, verrucarin Fumonisins: FB1, FB2 and FB3 Enniatins: ENNA, ENNA1, ENNB and ENNB1 BEA Zearalenone and metabolites: ZEN, ZAN, α-ZAL, α-ZEL, β-ZAL and β-ZEL DAS Fusaric acid Fusarin C Moniliformin	Trichothecenes: cereal products, rice, peanuts & maize (Sliwi, 2021). Fumonisins: cereal products, rice, asparagus, garlic, barley foods, beers, dried figs, milk, maize & black tea (Pierron et al., 2016b). Enniatins & BEA: cereal products, nuts, dried fruits & bananas (EFSA, 2014). ZEN: Wheat, barley, maize, sorghum, rye, rice, corn silage, sesame seed, hay, flour, malt, soybeans, beer, and corn oil (Ropejko and Twarużek, 2021). DAS: cereal products (Knutsen et al., 2018). Fusaric acid: maize & tomatoes (Han et al., 2014, Ismaiel and Papenbrock, 2015, Streit et al., 2013). Fusarin C: maize (Han et al., 2014). Moniliformin: cereal products(EFSA, 2017).
*Penicillium* spp. *Aspergillus* spp.	Ochratoxins: OTA A, B and C Cyclopiazonic acid Penicillic acid	Ochratoxins: cereal products, coffee, vegetables, liquorice, raisins, wine, nuts, pork & poultry (Ostry et al., 2013, Zorzete et al., 2013). Cyclopiazonic acid: maize, peanuts and cheese (Chrevatidis, 2003). Penicillic acid: cereal products (Ismaiel and Papenbrock, 2015).
*Aspergillus* spp. *Monascus* spp. *Penicillium* spp.	Citrinin	Cereal products, rice, pomaceous fruits, black olives, roasted nuts, spices, cheese (Ostry et al., 2013).
*Alternaria* spp.	Alternaria toxins: AME, AOH, TEA, ALT, ATX, TX	Small grain cereals, nuts, tomato fruits, olives, bell peppers, apples, berries, citrus fruits (Patriarca, 2016).
*Penicillium* spp.	Cyclochlorotine Luteoskyrin Rugulosin Patulin Roquefortine C	Cyclochlorotine: ereal products, soybeans, peanuts, beans (Otero et al., 2020). Luteoskyrin & rugulosin: rice (Mizutani et al., 2009). Patulin: apple and apple-based products, occasionally in other fruits such as pears, grapes, oranges and cereal products (Mahato et al., 2021b). Roquefortine C: carbonated beverages, beer, wine, meats, blue cheese and bread (Nicholson, 2004).
*Claviceps* spp.	Ergot alkaloids: ergometrine, ergocornine, ergocristine, ergotamine, ergosine and ergocryptine	Cereal products (Krska and Crews, 2008).
*Phomopsis* spp.	Phomopsin	Lupin seeds and products (EFSA, 2012b).

Note: AFB1, B2, G1, G2 = aflatoxin B1, B2, G1, G2; AFM1 = aflatoxin M1; AME = alternariol monomethyl ether; AOH = alternariol; ALT = altuene; ATX = altertoxin; BEA = beauvericin; CIT = citrinin; DAS = diacetoxyscirpenol; DON = deoxynivalenol; DOM-1 = de-epoxy-deoxynivalenol; 3/15-ADON = 3/15-acetyl deoxynivalenol; ENNA, A1, B, B1 = enniatins A, A1, B, B1; FB1, B2, B3 = fumonisin B1, B2, B3; HT2 = HT2-toxin; NIV = nivalenol; T2 = T2-toxin; TEA = tenuazonic acid; TX = tentoxin; OTA A, B, C = ochratoxin A, B, C; ZEN = zearalenone; ZAN = zearalanone; α-ZAL = α-zearalanol; α-ZEL = α-zearalenol; β-ZAL = β-zearalanol; β-ZEL = β-zearalenol.

### 3.2 Method planning

This protocol was developed based on the Conduct of Systematic Reviews in Toxicology and Environmental Health Research (COSTER) recommendations, covering 70 guidelines for good practice in the conduct of SRs, considering utility, transparency and credibility (Whaley et al., 2020). The project has been documented in an open-access record on the OSF platform (https://osf.io/9zdpj/), to ensure transparency in the development of this protocol. Additionally, the SR has also been registered in the International Prospective Register of Systematic Reviews or PROSPERO (CRD42022366592, https://www.crd.york.ac.uk/prospero). The goal of this process is to encourage collaborative and transparent practices in the SR of evidence.

### 3.3 Review team

A highly qualified and multidisciplinary team was chosen for this SR, consisting of scientists from Ghent University with particular expertise in mycotoxicology, human health, nutrition and SR methodologies. Each author was assigned a specific role based on their expertise (Table S6). T. Goessens has been responsible for the overall conduct and integrity of the review (*i.e.* guarantor of the review). For optimal project communication and management strategy, monthly meetings were established to aid in the preparation of the review protocol and syntax, and to assess the monthly progress.

### 3.4 Setting the research question

To evaluate the association between dietary mycotoxin exposure and adverse health effects in human populations, a research question was formulated using the PECO framework, and building on the research questions established during scoping (Table 1) (Morgan et al., 2018, Roth et al., 2020), resulting in the following: In individuals of all ages and genders (Population), what impact does dietary mycotoxin exposure (Exposure) have on human health (Outcome), in comparison to either no exposure or a reduced level of exposure (Comparator)?

#### 3.4.1 Population

Over the past years, various population studies have been conducted assessing the impact of dietary or non-dietary exposure of mycotoxins on human health (De Ruyck et al., 2020, Meerpoel et al., 2021, Viegas et al., 2019, Wallin et al., 2015). Within this SR, human populations of all age, gender, health status and life stage at dietary exposure or outcome assessment will be included. Potential confounding in the above mentioned characteristics will be acknowledged during evidence synthesis and accounted for when assessing the quality of evidence. Animals and human organs, tissues, cell lines or cellular components, as well as occupational, non-dietary exposed populations (*e.g.* occupational exposure resulting in sick building syndrome or pulmonary mycotoxicosis) will be excluded.

#### 3.4.2 Exposure

Overall, the main sources of dietary exposure to mycotoxins are contaminated crops, processed foods made from contaminated crops, and animal products from animals fed mycotoxin-contaminated feed (Nicholson, 2004). Most fungi-producing mycotoxins, such as *Fusarium* spp. and *Alternaria* spp. infect crops prior to harvest, whilst others, such as *Aspergillus* spp. and *Penicillium* spp., become active after harvest during inappropriate storage conditions, *i.e.* high humidity, high temperature and poor ventilation. Once contaminated crops are processed into food products such as bread, pasta and peanut butter, mycotoxins can end up in the final product. Additionally, animals that consume contaminated feed can accumulate mycotoxins in their tissues, which can then be passed on to humans through consumption of meat, milk, and other animal products (Nicholson, 2004). Based on a thorough scoping exercise, 69 mycotoxins with dietary importance were selected. As illustrated within Table 1, one fungal species may produce numerous different mycotoxins, and the same mycotoxin may also be produced by several dissimilar species.

#### 3.4.3 Comparator

In cohort studies and cross-sectional studies, health outcomes will be compared amongst groups having different levels of dietary exposure, assessed through environmental and consumption data, biomonitoring studies or physiologically based pharmacokinetic modelling. In randomized controlled trials and case-control studies, control groups will be defined as not receiving the tested oral mycotoxin(s). To ensure coverage of all available data on dietary mycotoxin-related health outcomes, non-controlled trials and case reports will be included into the SR as well, however being rated down accordingly during quality appraisal.

#### 3.4.4 Outcome

Mycotoxins have the potential to contribute to a diversity of adverse human health outcomes in humans, with the foremost toxic effects pertaining to their carcinogenicity, nephrotoxicity, hepatotoxicity, oestrogenicity, neurotoxicity and immunosuppression (Peraica et al., 1999b). The International Agency for Research on Cancer (IARC) has classified several mycotoxins according to their carcinogenicity to humans (Table S7 of Supplementary Material) (IARC, 2021). During scoping, a total of 15 dietary mycotoxin-related adverse health effects were prioritized. In order to construct a more precise and targeted search query, and to avoid potential errors related to the extent of the search, these keywords/health effects were divided into 3 major categories, *i.e.* cancer (1), non-carcinogenic diseases (2) and reproductive and developmental adverse outcomes (3). A discrepancy was made between prioritized primary outcomes (*i.e.* all types of cancer, kidney disease, liver disease, immunosuppression, gastrointestinal disease, inflammatory bowel syndrome, alimentary toxic aleukia, endocrine system disease, metabolic syndrome, Parkinson’s disease, Alzheimer’s disease, reproductive toxicity, developmental problems and cardiovascular disease) and secondary outcomes (*i.e.* microbiota alterations and clinical symptoms). Effects on chromatin (*i.e.* DNA mutations, gene expression, RNA & histone modifications) will not be included since this is out of the scope of the current SR. Furthermore, as the objective of this review aims at studying the mycotoxin-related health outcomes, studies of the levels of certain proteins and cell types, as well as studies on physiological (*e.g.* blood pressure, body mass index) or biochemistry parameters, without the diagnosis of a specific health outcome, will not be included.

#### 3.4.5 Study design

Adverse health effects of mycotoxins have been extensively studied in animals (Furian et al., 2022), *in vitro* (Skrzydlewski et al., 2022), *in silico* (Dellafiora et al., 2018) and even *ex vivo* (Kolf-Clauw et al., 2013). Although such studies are useful to provide an initial insight into the toxic potential of the mycotoxin, they lack a complex biological context (*in vitro*, *in silico* and *ex vivo* studies), or are affected by species differences (animal studies) impacting the trustfulness of extrapolation. As such, both human observational studies (*i.e.* cross-sectional, cohort & (non–)case-control), as well as intervention studies (*i.e.* clinical trials) have been proposed as top tools to explore associations between exposure to chemicals and health outcomes (Rosenbaum, 2009), and animal & mechanistic studies will only be integrated into the discussion section of this SR. Although intervention studies can provide valuable information on the dose–response relationship between mycotoxin exposure and health outcomes, their conduct remains limited since it involves exposing individuals to different levels of mycotoxins below the threshold of toxicological concern, implying the need for highly sensitive detecting techniques and raising ethical concerns when pertaining to mycotoxins having carcinogenic potential (Vidal et al., 2018). As such, observational studies have been the method of choice to study mycotoxin-related health effects in humans. Finally, despite the fact that case reports have limited generalizability, results can provide valuable information on rare or unusual health outcomes and results should at least be reported when constructing a SR, being however accordingly appraised (Vandenbroucke, 2001).

## 4 Search strategy

### 4.1 Information sources

#### 4.1.1 Bibliographic databases

The following databases were selected and a customized search string was constructed for each:

• Embase

This is a bibliographic database that covers the fields of biomedicine, pharmacology, and nursing. It contains over 30 million records, including abstracts and citations, from over 8,500 international biomedical journals. The database is updated on a daily basis and spans the years 1947 to the present. It also contains records from conference proceedings, dissertations, and other sources of grey literature (Elsevier, 2023a).

• PubMed

This database contains over 35 million citations and abstracts for biomedical literature. It includes journal articles, books, and other types of scientific and medical literature, such as conference proceedings and dissertations. PubMed covers a wide range of topics, including (epi)genetics, environmental, and public health (National Library of Medicine, 2023).

• Cochrane Library

The Cochrane Library's evidence-based medicine databases provide high-quality, up-to-date information on the effects of interventions for many health conditions. It contains several databases, including the Cochrane Database of SRs, which contains SRs and *meta*-analyses of healthcare interventions. The Cochrane Methodology Register and the Cochrane Central Register of Controlled Trials (CENTRAL) are also part of the library (John Wiley & Sons, 2023).

• Web of Science

The Web of Science citation database provides a multidisciplinary coverage of over 10,000 high-impact journals in the sciences, social sciences, and arts and humanities, as well as international proceedings coverage for over 120,000 conferences (Clarivate, 2023). Within this SR, specifically the Web of Science Core Collection was searched.

• Scopus

Scopus uniquely combines a comprehensive, expertly curated abstract and citation database with enriched data (>90 curated documents) and linked scholarly literature across a wide variety of disciplines (Elsevier, 2023b).

#### 4.1.2 Bibliographic references

To ensure retrieval of all relevant human studies, bibliographic references in the papers selected for final analysis will be additionally screened, as described in *3.4 Citation tracking*.

### 4.2 Search string

For each health outcome category, search strings were developed based upon the scoping exercise in section 3.1, followed by a further exploration of the health outcome-related keywords using the MeSH and Emtree databases (1), predefined search blocks (BioMedical information, 2020) (2), and previously published search strings (3) (Claeys et al., 2020, Tesfamariam et al., 2019), as detailed in Fig. S1 of Supplementary Material. Predefined search blocks were found for cancer, kidney disease, inflammatory bowel disease, metabolic syndrome, Parkinsonian disorders, Alzheimer disease, cardiovascular disease and altered microbiota, and can be consulted at https://blocks.bmi-online.nl/ (BioMedical information, 2020). Each detailed electronic search string per bibliographic database (Pubmed, Embase,Cochrane Library, Web of Science and Scopus) is presented in Table S8, S9, S10, S11 and S12 of Supplementary Material, respectively.

### 4.3 Data management

**Fig. 1:**
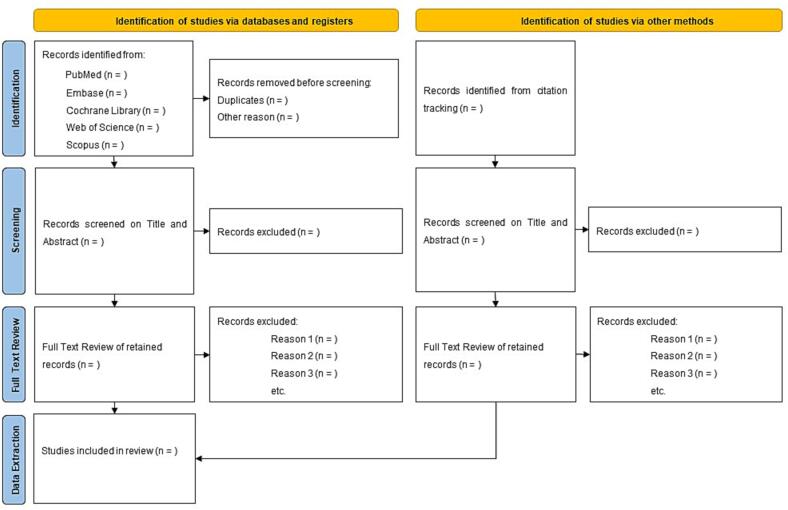
PRISMA 2020 flow diagram adapted to the current systematic review protocol (Page et al., 2021).

All references retrieved from PubMed, Cochrane Central, Embase, Web of Science Core Collection and Scopus will be imported to Covidence (www.covidence.org), either as a PubMed or RIS text format. Within Covidence, all duplicates will be automatically removed, and *Screening on Title and Abstract* will be performed independently by two different reviewers (TG and YB). Only studies that meet the set criteria will be considered for *Full Text Review*. If there is any disagreement, it will be resolved by a third reviewer (two-third majority). Finally, data from retained eligible studies will be extracted and exported to data extraction forms in MS Excel (*Data Extraction*), by the principal reviewer (TG), and peer-checked by a second reviewer (FV). The data extraction forms are publicly available via https://osf.io/9zdpj/. All extracted information (*i.e.* primary and secondary outcomes, as well as other types of variables such as population characteristics and study design) will be included in the SR report. A PRISMA flowchart detailing each stage of the selection procedure and any disqualifying factors will be included in the SR report, as required by the PRISMA statement (Fig. 1) (Page et al., 2021). Furthermore, updates to the search, aiming to expand the evidence base through time (*i.e.* publication filters), will involve repeating the original process of review conduct, and writing an updated version including the new evidence, updated PRISMA workflow & conclusions. In case protocol amendments will be necessary (*e.g.* a change in methods, a correction to the original report or the inclusion of additional evidence without an updated search), a new protocol for submission will be constructed, specifying the changes as ‘modifications’ to the registered protocol (Bayliss et al., 2016).

### 4.4 Citation tracking

In order to ensure that all relevant studies will be included in our analysis, we will conduct a thorough examination of the cited and citing literature for all selected studies, using a specialized tool, *i.e. citationchaser* (https://estech.shinyapps.io/citationchaser/), compiling a comprehensive and deduplicated list of cited and citing records and following the same workflow as described above, *i.e. Screening on Title and Abstract*, *Full Text Review* and *Data Extraction*. The latter allows us to identify any additional eligible studies that may have been missed during the initial electronic searches and ensures that the analysis is as comprehensive as possible.

### 4.5 Plan for re-running searches

One month prior to submission, a re-run of our search will be performed, limiting those articles to the latest month or year, to ensure inclusion of up-to-date eligible studies. In the subsequent searches, each database will be searched using the same search strings as in the first run, following the same approach as described above (*Screening on Title and Abstract, Full Text Review* and *Data Extraction*).

## 5 Study selection

### 5.1 Inclusion & exclusion criteria

The PECO statement was used to develop eligibility criteria for the SR (Morgan et al., 2018, Schreier et al., 2022). Human studies (*i.e.* cohort, cross-sectional studies, (non–)case-control, case reports and clinical trials, both randomized and non-randomized) that contain information on one or more dietary mycotoxins as well as adverse human health outcomes (*i.e.* primary outcomes pertaining to cancer, non-carcinogenic diseases and reproductive & developmental adverse outcomes, as well as secondary outcomes such as clinical symptoms and microbiota alterations) will be included. All published primary research articles, except for reviews and expert’s opinions, will be included in the indexed scientific literature. Secondary literature as well as conference abstracts, presentations, posters, book chapters, and theses/dissertations are excluded. There will be no restriction on publication years or written languages. While animal and mechanistic (both animal and human) studies can provide valuable insights into mechanism of action and potential effects of interventions, their limitations and differences from human physiology necessitate cautious interpretation (*i.e.* translational relevance). By excluding these types of study, the robustness of evidence-based conclusions specifically for human health is guaranteed (Van Norman, 2019). Nevertheless, to elucidate the underlying biological mechanisms of action, all findings of the SR will be thoroughly discussed integrating evidence derived from both animal studies and mechanistic investigations. This will involve a comprehensive search & analysis of the existing literature using the pinpointed mycotoxin & outcome, as well as the following keywords: *in vitro*, cell line, culture, *in vivo*, animal (model/study/trial/experiment), organism, preclinical, *ex vivo*, organoid, mechanistic, mechanism, pathway analysis and molecular signaling (BioMedical information, 2020). All inclusion and exclusion criteria of this SR according to the PECO framework are presented in Table 3.

**Table 3:** Inclusion and exclusion criteria of the SR according to the PECO framework.

**Inclusion criteria**	**Exclusion criteria**
***Population***	
Human - No restrictions on age, gender, health status or life stage at exposure or outcome assessment. Only whole organisms are considered.	Animals and human organs, tissues, cell lines or cellular components.
***Exposure***	
Dietary exposure of mycotoxins, either measured (*e.g.* environmental and consumption data, or biomonitoring studies) or modelled (*e.g.* physiologically based pharmacokinetic modelling).	Non-dietary routes (*e.g.* inhalation, dermal).
***Comparator***	
Human - in cohort studies and cross-sectional studies, health outcomes will be compared amongst groups having different levels of dietary exposure, assessed through environmental and consumption data, biomonitoring studies or physiologically based pharmacokinetic modelling. In randomized controlled trials and case-control studies, control groups will be defined as not receiving the tested oral mycotoxin(s).	Animals and human organs, tissues, cell lines or cellular components.
***Outcome***	
All adverse health outcomes (primary outcomes divided into 3 categories *i.e.* cancer, non-carcinogenic diseases and reproductive & developmental adverse outcomes, as well as secondary outcomes) measured in the exposed humans.	Effects on chromatin (*i.e.* DNA mutations, gene expression, RNA & histone modifications).
***Study design***	
Cohort studies, (non-)randomized controlled trials, (non-)case-control studies, cross-sectional studies and case reports.	*In vitro*, *ex vivo*, *in silico* and review studies, as well as expert’s opinions.

### 5.2 Selection process

Following the automatic deduplication described in section 4.3, screening on *Title and Abstract* will be performed independently by two different reviewers (TG and YB) and only studies that meet inclusion criteria elements will be considered for Full Text Review. If there is any disagreement, it will be resolved by a third reviewer (MDB), *i.e.* two-third majority. A detailed overview of the selection process will be presented in a PRISMA flowchart.

### 5.3 Pilot study

Before reviewing the full-text of all of the retrieved articles, a pilot test of the review process and the eligibility criteria was conducted on a small sample of the retrieved articles. Insights from this study were used to establish a standardized, routine review process, and modify the criteria in the data extraction form, ensuring that in the full SR the most relevant information is extracted from all the papers in one phase, in a uniform, objective way, without the need to retrieve individual papers at a later stage (Long, 2014). As such, the authors have conducted a pilot study using the search strategy described in section 4. A *Screening on Title and Abstract* was performed independently by TG and YB on 1,500 articles based upon the predefined eligibility criteria and PECO elements. Disagreements were solved by MDB, *i.e.* two-third majority, and the justification was documented in Covidence as a comment tag linked to the article. Only studies that met the set criteria were considered for *Full Text Review* (n = 35) and the justification for exclusion was documented in MS Excel worksheets (https://osf.io/9zdpj/). During the *Full Text Review*, discussions were held between all authors (TG, TMM, KT, FV, YB, SDS, CL and MDB) and it was decided to not include mycotoxin-induced DNA, RNA or histone modifications as a health outcome into the SR process since this was out of the scope of the current SR (also see section 3.4 Setting the Research Question). To ensure qualitative extraction and avoid subjective classifications, a data extraction form was created to be implemented in Covidence, with clearly defined questions, examples, and predefined answer options where possible, which can be consulted at https://osf.io/9zdpj/. A calibration exercise was set up to ensure that all reviewers understood the data extraction forms, aiming to reduce discrepancies between reviewers. Finally, retained eligible studies (n = 5) were extracted in Covidence by TG and peer-reviewed by FV (*Data Extraction*), and results were exported to MS excel worksheets (https://osf.io/9zdpj/). During this process, and in open dialogue with all reviewers, extraction parameters were further fine-tuned, implementing the specification of mycotoxin biomarkers of exposure (*e.g.* aflatoxin albumin adduct) and narrowing the analytical methodology details solely to the applied technique, *e.g.* liquid chromatography tandem mass spectrometry (LC/MS-MS), rather than the instrument specification details. Finally, the quality of evidence was evaluated for each of the 5 included studies. Overall, this pilot study has elucidated the eligibility criteria for reviewers and facilitated the efficiency, as well as ensured the objectivity of the review process and data extraction, which will increase the validity of the full SR.

## 6 Evidence synthesis

### 6.1 Data extraction

Data extraction forms were developed in Covidence using the fundamental principles of the Cochrane Handbook for Systematic Reviews of Interventions (Glenton et al., 2022). Extraction parameters were constructed based upon (1) the Cochrane Collaboration's “Data collection form for intervention review - RCTs and non-RCTs” template (Effective Practice and Organisation of Care (EPOC), 2013), and (2) previous mycotoxin-health-related SRs (Claeys et al., 2020, Tesfamariam et al., 2019), whilst taking into account the primary research question and PECO elements (section 3.4), to cover the following data: general article information, study design characteristics, population characteristics, mycotoxin measurement technologies, health outcome specifics, results of the study (both primary and secondary research outcomes) and statistical approach. The general data extraction form can be found at https://osf.io/9zdpj/.

### 6.2 Presenting the evidence

**Fig. 2:**
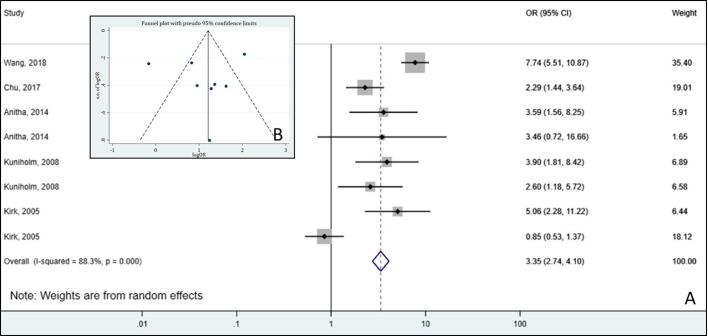
Forest (A) and funnel plot (B) of aflatoxin exposure and risk of liver cirrhosis using odds ratios. Adapted from Nigussie Mekuria et al. (2020) (Nigussie Mekuria et al., 2020).

Based upon the scoping-identified heterogeneity among mycotoxin-exposure studies in terms of measured mycotoxins, selected health outcomes, variation in measurement method and matrix analysis, failing to adjust to confounders (Tesfamariam et al., 2019), and differences in study design, a narrative approach is deemed highly suitable for evidence synthesis (Roberts et al., 2006). A detailed summary of the included studies, their characteristics, and the outcomes of interest will be provided within a tabular format, as presented in Table S13 of Supplementary Material. Based upon the expected variables of heterogeneity, the included studies will be clustered according to the studied mycotoxin (1) and health outcome (2), and further subgrouped according to the study design (3) and analyzed matrix (4). If a *meta*-analysis is deemed applicable (section 6.3), forest or funnel plots will be used to graphically display and explore potential relationships (Fig. 2). Additionally, heat maps will be constructed and accordingly discussed to provide information on available knowledge and knowledge gaps concerning studied health outcomes per dietary mycotoxin. Finally, the reported mycotoxin-related health risk per adverse health outcome, will be discussed, together with the quality of evidence, the latter provided as a summary of the performed RoB and certainty assessment as described in section 6.1 (Robvis Visualisation Tool, 2022).

### 6.3 Statistical analysis

If it is determined that the different research outcomes cannot be combined based on differences in elements of the predefined PECO research question, variation in the way confounding is considered in the analysis, or risk of other types of biases, all the results and characteristics of the individual studies will be displayed in a descriptive, qualitative, form (Glenton et al., 2022, Tesfamariam et al., 2019). If studies have similar designs and study populations, and the study outcome is measured in a similar way, study findings will be combined, and a *meta*-analysis will be performed, based on tests for heterogeneity of effect sizes. The effect measures that we plan to use for dichotomous outcomes are Odds Ratio (OR) and Risk Ratio (RR), whereas for continuous outcomes we plan to use the Mean Difference (MD), the Standardized Mean Difference (SMD) and the Correlation Coefficient (CC). To make the effect sizes comparable across the included studies, we covert them into common metric using mathematical formulas, taking into account certain levels of equivalence among various measures of effect size (Borenstein et al., 2009). Heterogeneity will be assessed by means of a forest plot, Cochran’s Q test (χ^2^ test) or I^2^ index (Ahn and Kang, 2018). If the heterogeneity is low (*e.g.* I^2^ index close to 0 – 25 %), a fixed-effects model will be appropriate to combine data because all included studies are functionally identical and the observed variation is mostly due to chance. In cases of moderate heterogeneity, a random-effects model with RevMan software will be used when it assumes that the true effect sizes only vary between studies (Borenstein et al., 2010, RevMan Version 5, 2023). For significant heterogeneity, *i.e.* p-value < 0.1 (χ^2^ test) or I^2^ index > 75 %, we will perform *meta*-regression analysis using a mixed-effects model in R’s metafor package to investigate how study-level characteristics (*e.g.* study design, population, sample size, year of publication or specific methodological features) might relate to the effect sizes. This model accounts for the variability at different levels of hierarchical data and data non-independence by integrating fixed effects for within-in group variation and random effects for between-group differences. If the heterogeneity is too high and inexplicable, we will shift toward a qualitative synthesis rather than a quantitative meta- analysis.

Lastly, publication bias, *i.e.* distortion of *meta*-analysis outcomes due to the higher likelihood of publication of statistically significant studies rather than non-significant studies, will be assessed using Egger’s regression and funnel plots (Shi et al., 2019), followed by the trim-and-fill test to visualize potentially missing data, and statistically both detect and correct for funnel asymmetry.

### 6.4 Sensitivity analysis

We will only carry out a sensitivity analysis on studies considered to be at a low RoB, as elaborated in section 7.1. When sensitivity analyses show that the overall result and conclusions of the SR are not affected by the different decisions made during the review process, the results of the review will be appraised with a higher degree of certainty. Where sensitivity analyses identify particular decisions or missing information greatly influencing the findings of our SR, efforts will be made to resolve uncertainties and obtain additional information (*e.g.* contacting trial authors), and if unsuccessful, results will be interpreted with an appropriate degree of caution (Glenton et al., 2022, Mcgrath et al., 2018). Finally, the impact of clustering or data non-independence, differential mycotoxin analysis (*e.g.* different limit of detection to identify mycotoxins among labs and analyzing techniques) and multiple risks with composite outcomes (*e.g.* aflatoxin-exposed cancer patients having diabetes) will be assessed (Truong et al., 2022).

## 7 Quality of evidence

### 7.1 Risk of bias

To assess the internal validity of the included studies in our own review, a risk of bias (RoB) assessment will be performed based on the Focus-Extent-Application-Transparency (FEAT) criteria (Frampton et al., 2022). Two reviewers (TG and YB) will conduct the primary RoB and a third reviewer (MDB) will resolve potential conflicts (*i.e.* two-third majority). All authors (TG, TMM, KT, FV, YB, SDS, CL and MDB) will review the assessment and may raise any points of concern to reach a final majority-based decision. The outcomes of the RoB assessment are pivotal in establishing the reliability and credibility of the studies incorporated in the SR. They enable the identification of potential sources of bias or confounding that might have influenced the study findings. To assess the RoB, we will use the Office of Health Assessment and Translation of the National Toxicology Program (OHAT) RoB rating tool (https://ntp.niehs.nih.gov/go/riskbias. This tool allows to evaluate the RoB in different human studies, making it easier to consider different types of evidence streams, using general applicable terms and categories. Specifically, appraisal is performed by means of 11 questions, grouped under 6 types of potential sources of bias: selection, confounding, performance, attrition/exclusion, detection, and selective reporting (see Table 4). For each RoB question, the tool requires reviewers to select between low and high RoB options. More specifically, there are 2 low-risk options: “definitely low”, which indicates there is direct evidence of low RoB practices, and “probably low”, indicating that there is indirect evidence of low RoB practices OR it is deemed that deviations from low RoB practices for these criteria during the study would not appreciably bias results, including consideration of direction and magnitude of bias. There are also 2 high-risk options: “probably high”, pertaining to the fact that there is indirect evidence of high risk of bias practices OR there is insufficient information provided about relevant RoB practices (*e.g.* not reported or “NR”), and “definitely high”, if there is direct evidence of high RoB practices. For each question in the OHAT tool, one of four answers will be selected following the OHAT instructions. For each study, RoB is assessed at the outcome level because certain aspects of study design and conduct may increase the RoB for some outcomes but not others within the same study (Eick et al., 2020). The general instruction format of the OHAT RoB rating tool and the specific questions are presented in Table 3. After answering each question, a justification will be provided in a separate text field. This justification will detail the design, conduct, or observations that support the decision, as well as any points of concern raised during the final decision making. All of this information will be thoroughly documented in Microsoft Excel sheets. Finally, two distinct outputs will be generated pertaining to the RoB. The initial output will take the form of a graphical representation as a weighted bar plot that delineates the distribution of studies based on their corresponding bias judgments using the Robvis visualization tool (Robvis Visualisation Tool, 2022). The second output will take the form of a written summary that provides a comprehensive overview of the RoB present within the study. Information regarding the decision process and potential conflicts will be presented as supplementary material.

**Table 4:** Format of the OHAT RoB rating tool (NTP, 2019).

***Bias Domains and Questions***					
• 11 Risk of bias questions or domains • Each question is applicable to 1 to 6 study design types • Questions are rated by selecting among 4 possible answers • Questions are grouped under 6 types of bias (selection, confounding, performance, attrition/exclusion, detection, and selective reporting) • In practice, web-based forms will be used and reviewers will only see questions and instructions that are relevant to the study under review	**Controlled Trial**	**Cohort study**	**Case-control study**	**Cross-sectional study**	**Case Report**
***Selection Bias***					
1. Was administered dose or exposure level adequately randomized?	**X**				
2. Was allocation to study groups adequately concealed?	**X**				
3. Did selection of study participants result in appropriate comparison groups?		**X**	**X**	**X**	
***Confounding Bias***					
4. Did the study design or analysis account for important confounding and modifying variables?		**X**	**X**	**X**	**X**
***Performance Bias***					
5. Were experimental conditions identical across study groups?	**X**				
6. Were the research personnel and human subjects blinded to the study group during the study?	**X**				
***Attrition/Exclusion Bias***					
7. Were outcome data complete without attrition or exclusion from analysis?	**X**	**X**	**X**	**X**	
***Detection Bias***					
8. Can we be confident in the exposure characterization?	**X**	**X**	**X**	**X**	**X**
9. Can we be confident in the outcome assessment?	**X**	**X**	**X**	**X**	**X**
***Selective Reporting Bias***					
10. Were all measured outcomes reported?	**X**	**X**	**X**	**X**	**X**
***Other Sources of Bias***					
11. Were there no other potential threats to internal validity (*e.g.* statistical methods were appropriate and researchers adhered to the study protocol)?	**X**	**X**	**X**	**X**	**X**

### 7.2 Certainty assessment

**Fig. 3:**
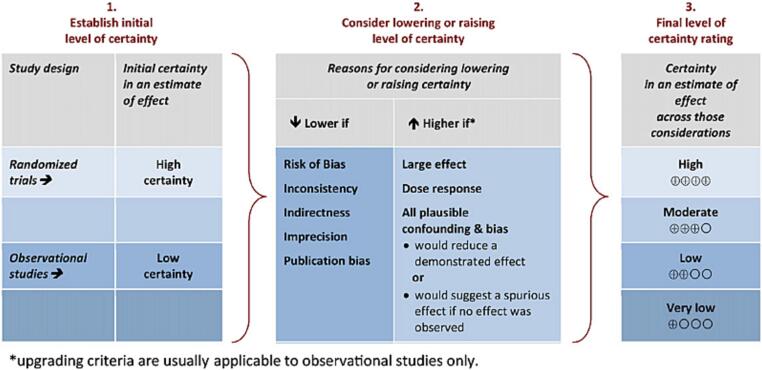
GRADE’s approach to develop certainty ratings across a body of evidence for each outcome based on a systematic review and across outcomes (Morgan et al., 2016).

To provide a clear and concise summary of the overall certainty of evidence, we will use the Grading of Recommendations Assessment, Development and Evaluation (GRADE) approach (Morgan et al., 2016). An initial evaluation of the evidence’s certainty is based on whether or not the research studies used randomized allocation. In the current GRADE approach, the randomized controlled trials (RCT) receive an initial rating of “high”, whereas observational (*i.e.* non-randomized) studies start at “low”. After this initial evaluation, the certainty in a body of evidence can be rated down for RoB (section 7.1), inconsistency (*i.e.* minimal or no overlap of confidence intervals or wide variance of point estimates across studies), indirectness (*i.e.* comparison of different populations, mycotoxin(s) exposures or health outcomes), imprecision (*i.e.* number of included participants lower than the appropriate sample size calculation), or publication bias, the latter evaluated by tests for asymmetry of funnel plots. Finally, the evidence can be rated up for the magnitude of the effect (Table 5), the presence of a clear dose–response gradient and the impact of residual plausible confounding (*i.e.* whether or not comparison groups were matched) (Morgan et al., 2016). The GRADE’s approach to developing certainty ratings across a body of evidence has been illustrated in Fig. 3. Again, each study will be evaluated by two independent reviewers (TG and YB), and a third reviewer (MDB) will resolve potential conflicts (*i.e.* two-third majority). The result will be reviewed by all authors (TG, TMM, KT, FV, YB, SDS, CL and MDB) and each conflict influencing the final majority-based decision will be thoroughly documented in MS excel worksheets to be consulted as supplementary material.

**Table 5:** Rating up evidence for magnitude of effect using the GRADE approach for certainty assessment (Morgan et al., 2016).

RR, OR*		
Magnitude of effect	Definition	Quality of evidence
Large	RR* >2 or < 0.5 (based on direct evidence, with no plausible confounders)	may increase 1 level
Very large	RR* >5 or < 0.2 (based on direct evidence with no serious problems with risk of bias or precision, *i.e.* with sufficiently narrow confidence intervals)	may increase 2 levels
**Other outcome measures**		
One may be more likely to rate up the quality of evidence because of large or very large magnitude of an effect, when: • effect is rapid • effect is consistent across subjects • previous trajectory of disease is reversed • large magnitude of an effect is supported by indirect evidence		

Note: * = for evaluating the magnitude of an effect based on OR, it is suggested to first convert OR to RR.

## 8 Conclusion

This protocol describes the methodology for the conduct of a SR on mycotoxin-related human health risks. Based upon an extensive scoping exercise, and taking into account Population, Exposure, Comparator, Outcome (PECO) and study design, a research question and corresponding search string was developed and a pilot study was conducted. A detailed, in-depth approach has been presented for evidence synthesis, including the clustering and subgrouping of data, and quality assessment. The resulting review will identify and appraise all mycotoxin-health related literature to date, whilst providing a broader view of the remaining knowledge gaps and areas of uncertainty. Any changes made to the original registered protocol will be documented to maintain review integrity. This research aims to steer future research and risk assessment activities (*e.g.* population-based cohort studies) to support evidence-based decision- and policy-making.

## Funding

This research was funded by the European Research Council (ERC) under the European Union’s Horizon 2020 research and innovation program (grant agreement No 946192, HUMYCO).

## CRediT authorship contribution statement

**T. Goessens:** Conceptualization, Data curation, Methodology, Validation, Visualization, Investigation, Writing – original draft, Project administration. **T. Mouchtaris-Michailidis:** Conceptualization, Methodology, Writing – review & editing. **K. Tesfamariam:** Conceptualization, Writing – review & editing. **N.N. Truong:** Writing – review & editing. **F. Vertriest:** Writing – review & editing. **Y. Bader:** Writing – review & editing. **S. De Saeger:** Conceptualization, Writing – review & editing. **C. Lachat:** Conceptualization, Writing – review & editing. **M. De Boevre:** Conceptualization, Validation, Supervision, Writing – review & editing, Project administration, Funding acquisition.

## Declaration of competing interest

The authors declare that they have no known competing financial interests or personal relationships that could have appeared to influence the work reported in this paper.

## Data Availability

I have shared the link to my data in the protocol.
