# Supplementary material for: Dietary mycotoxin exposure and human health risks: A protocol for a systematic review
Source: Environ Int. 2024 Feb;184:108456. doi: 10.1016/j.envint.2024.108456 (PMC10895515; doi:10.1016/j.envint.2024.108456)
Supplement: Supplementary data 1 [file mmc1.docx]

Dietary mycotoxin exposure and human health risks:

a protocol for a systematic review

T. Goessens^1*^, T. Mouchtaris-Michailidis^1*^, K. Tesfamariam^2^, N.N. Truong^1^, F. Vertriest^1,3^, Y. Bader^1^, S. De Saeger^1^, C. Lachat^2^, M. De Boevre^1^

^1^ Center of Excellence in Mycotoxicology and Public Health, Faculty of Pharmaceutical Sciences, Ghent University, Ghent, Belgium.

^2^ Department of Food Technology, Safety and Health, Faculty of Bioscience Engineering, Ghent University, Ghent, Belgium.

^3^Ghent University, Department of Internal Medicine and Pediatrics, Faculty of Medicine and Health Sciences, Ghent, Belgium

*shared first author.

**TABLE OF CONTENT**

**Table S1.** Detailed electronic search string for the **scoping exercise** in PubMed.

**Table S2.** Detailed electronic search string for the **scoping exercise** in Embase.

**Table S3.** Detailed electronic search string for the **scoping exercise** in Cochrane Library.

**Table S4.** Detailed electronic search strings for the **scoping exercises** in registry databases.

**Table S5.** Title, PECO elements, core methodology, objective and key conclusion of the 22 review articles withheld during scoping.

**Table S6.** Members of the review team who have been assigned to core SR competencies.

**Table S7.** Mycotoxins classified according to the IARC Monograph that identifies environmental causes of cancer in humans.

**Table S8.** Detailed electronic search string for the **systematic review** in PubMed.

**Table S9.** Detailed electronic search string for the **systematic review** in Embase.

**Table S10.** Detailed electronic search string for the **systematic review** in Cochrane Library.

**Table S11.** Detailed electronic search string for the **systematic review** in Web of Science.

**Table S12.** Detailed electronic search string for the **systematic review** in Scopus.

**Table S13.** Example of the tabular format covering the characteristics of the included studies and the outcomes of interest for inclusion into the systematic review.

**Figure S1.** Schematic overview of the search string development. MeSH = Medical Subject Headings, BMI = BioMedical Information (BioMedical information, 2020).

**Table S1.** Detailed electronic search string for the scoping exercise in PubMed.

| **Electronic search string – 300 results – no filters** |
| --- |
| Primary search string:  (3-acetyldeoxynivalenol[tiab] OR 15-acetyldeoxynivalenol[tiab] OR afla*[tiab] OR aspertoxin[tiab] OR “aspergillic acid”[tiab] OR “cyclopiazonic acid”[tiab] OR altertoxin[tiab] OR alternariol[tiab] OR “alternariol monomethyl ether”[tiab] OR alpha-zearalanol[tiab] OR alpha-zearalenol[tiab] OR altenuene[tiab] OR beauvericin[tiab] OR beta-cyclopiazonic acid[tiab] OR beta-zearalanol[tiab] OR beta-zearalenol[tiab] OR citrinin[tiab] OR cyclochlorotine[tiab] OR deoxynivalenol[tiab] OR vomitoxin[tiab] OR diacetoxyscirpenol[tiab] OR dihydro-O-methylsterigmatocystin[tiab] OR dihydroxy-aflavinine[tiab] OR dihydroxyl-O-methylsterigmatocystin[tiab] OR ditryptophenaline[tiab] OR enniatin*[tiab] OR “ergot alkaloid*”[tiab] OR flavacol[tiab] OR fumonisin*[tiab] OR “fusarenon X”[tiab] OR “fusaric acid”[tiab] OR “fusarin C”[tiab] OR “HT2 toxin”[tiab] OR “HT 2 toxin”[tiab] OR “hydroxyneoaspergillic acid”[tiab] OR “leporin C”[tiab] OR luteoskyrin[tiab] OR methylcitreo-isocoumarin[tiab] OR moniliformin[tiab] OR mycotoxin*[tiab] OR neosolaniol[tiab] OR nivalenol[tiab] OR ochratoxin*[tiab] OR O-methylsterigmatocystin[tiab] OR paspalinine[tiab] OR pathotoxin[tiab] OR patulin[tiab] OR phomopsin[tiab] OR “penicillic acid”[tiab] OR “roquefortine C”[tiab] OR rugulosin[tiab] OR speradine A[tiab] OR sterigmatocystin[tiab] OR “T2 toxin”[tiab] OR “T 2 toxin” OR “NX toxin”[tiab] OR tentoxin[tiab] OR “tenuazonic acid”[tiab] OR trichothecene*[tiab] OR verrucarin[tiab] OR versiconol[tiab] OR zearalenone[tiab] OR zearalanol[tiab] OR zearalenol[tiab] OR mycotoxin*[tiab] OR “fungal toxin*”[tiab] OR “mycotoxins”[MeSH Terms] OR “aflatoxins”[MeSH Terms] OR “ergot alkaloids”[MeSH Terms] OR “fusaric acid”[MeSH Terms] OR “alternariol”[Supplementary Concept] OR “alternariol monomethyl ether”[Supplementary Concept] OR “deoxynivalenol”[Supplementary Concept] OR “altertoxin”[Supplementary Concept] OR “fusarin C”[Supplementary Concept] OR “enniatins”[Supplementary Concept] OR “moniliformin”[Supplementary Concept] OR “versiconol”[Supplementary Concept] OR “zearalenol”[Supplementary Concept]) **AND** ("Systematic Review" [Publication Type] OR “systematic review”[tiab] OR "Meta-Analysis" [Publication Type] OR “meta-analysis”[tiab] OR “evidence map”[tiab] OR “systematic search”[tiab])  Secondary search string:  NOT (“Animals”[mesh] not “Humans”[mesh]) |

**Table S2.** Detailed electronic search string for the scoping exercise in Embase.

| **Electronic search string – 425 results – no filters** |
| --- |
| Primary search string:  (3-acetyldeoxynivalenol:ti,ab,kw OR 15-acetyldeoxynivalenol:ti,ab,kw OR afla*:ti,ab,kw OR aspertoxin:ti,ab,kw OR ‘aspergillic acid’:ti,ab,kw OR ‘cyclopiazonic acid’:ti,ab,kw OR altertoxin:ti,ab,kw OR alternariol:ti,ab,kw OR ‘alternariol monomethyl ether’:ti,ab,kw OR alpha-zearalanol:ti,ab,kw OR alpha-zearalenol:ti,ab,kw OR altenuene:ti,ab,kw OR beauvericin:ti,ab,kw OR ‘beta-cyclopiazonic acid’:ti,ab,kw OR beta-zearalanol:ti,ab,kw OR beta-zearalenol:ti,ab,kw OR citrinin:ti,ab,kw OR cyclochlorotine:ti,ab,kw OR deoxynivalenol:ti,ab,kw OR vomitoxin:ti,ab,kw OR diacetoxyscirpenol:ti,ab,kw OR dihydro-O-methylsterigmatocystin:ti,ab,kw OR dihydroxy-aflavinine:ti,ab,kw OR dihydroxyl-O-methylsterigmatocystin:ti,ab,kw OR ditryptophenaline:ti,ab,kw OR enniatin*:ti,ab,kw OR ‘ergot alkaloid*’:ti,ab,kw OR flavacol:ti,ab,kw OR fumonisin*:ti,ab,kw OR fusarenon X:ti,ab,kw OR ‘fusaric acid’:ti,ab,kw OR ‘fusarin C’:ti,ab,kw OR ‘HT 2 toxin*’:ti,ab,kw OR ‘HT2 toxin*’:ti,ab,kw OR ‘hydroxyneoaspergillic acid’:ti,ab,kw OR ‘leporin C’:ti,ab,kw OR luteoskyrin:ti,ab,kw OR methylcitreo-isocoumarin:ti,ab,kw OR moniliformin:ti,ab,kw OR mycotoxin*:ti,ab,kw OR neosolaniol:ti,ab,kw OR nivalenol:ti,ab,kw OR ochratoxin*:ti,ab,kw OR O-methylsterigmatocystin:ti,ab,kw OR paspalinine:ti,ab,kw OR pathotoxin:ti,ab,kw OR patulin:ti,ab,kw OR phomopsin:ti,ab,kw OR ‘penicillic acid’:ti,ab,kw OR roquefortine-C:ti,ab,kw OR rugulosin:ti,ab,kw OR ‘speradine A’:ti,ab,kw OR sterigmatocystin:ti,ab,kw OR ‘T2 toxin*’:ti,ab,kw OR ‘T 2 toxin*’:ti,ab,kw OR ‘NX toxin*’:ti,ab,kw OR tentoxin:ti,ab,kw OR ‘tenuazonic acid’:ti,ab,kw OR trichothecene*:ti,ab,kw OR verrucarin:ti,ab,kw OR versiconol:ti,ab,kw OR ‘versiconol hemiacetal acid’:ti,ab,kw OR zearalenone:ti,ab,kw OR zearalanone:ti,ab,kw OR mycotoxin*:ti,ab,kw OR ‘fungal toxin*’:ti,ab,kw OR ‘mycotoxin’/exp OR ‘aflatoxin’/exp OR ‘aflatoxin B’/exp OR ‘aflatoxin B1’/exp OR ‘aflatoxin B2’/exp OR ‘aflatoxin G1’/exp OR ‘aflatoxin G2’/exp OR ‘aflatoxin M1’/exp OR ‘alpha zearalenol’/exp OR ‘alternariol’/exp OR ‘beta zearalenol’/exp OR ‘diacetoxyscirpenol’/exp OR ‘fumonisin’/exp OR ‘fumonisin A1’/exp OR ‘fumonisin B1’/exp OR ‘fumonisin B2’/exp OR ‘fusarenon X’/exp OR ‘HT 2 toxin’/exp OR ‘luteoskyrin’/exp OR ‘masked mycotoxin’/exp OR ‘moniliformin’/exp OR ‘nivalenol’/exp OR ‘ochratoxin’/exp OR ‘patulin’/exp OR ‘sterigmatocystin’/exp OR ‘T 2 toxin’ OR ‘tenuazonic acid’/exp OR ‘trichothecene’/exp OR ‘verrucarin A’/exp OR ‘verrucarin A derivate’/exp OR ‘versicolorin A’/exp OR ‘vomitoxin’/exp OR ‘zearalenone’/exp OR ‘penicillic acid’/exp OR ‘cyclopiazonic acid’/exp OR ‘beauvericin’/exp OR ‘citrinin’/exp OR “enniatin’/exp OR ‘ergot alkaloid’/exp OR ‘fusaric acid’/exp OR ‘neosolaniol’/exp) **AND** (‘systematic review’/exp OR ‘systematic review’:ti,ab,kw OR ‘Meta analysis’/exp OR ‘meta-analysis’:ti,ab,kw OR ‘evidence map’:ti,ab,kw OR ‘systematic search’:ti,ab,kw)  Secondary search string:  NOT (‘animal’/exp not ‘human’/exp) |

**Table S3.** Detailed electronic search string for the scoping exercise in Cochrane Library.

| **Electronic search string – 3 results – no filters** |
| --- |
| Primary search string:  (3-acetyldeoxynivalenol:ti,ab,kw OR 15-acetyldeoxynivalenol:ti,ab,kw OR afla*:ti,ab,kw OR aspertoxin:ti,ab,kw OR aspergillic NEXT/1 acid:ti,ab,kw OR cyclopiazonic NEXT/1 acid:ti,ab,kw OR altertoxin:ti,ab,kw OR alternariol:ti,ab,kw OR alternariol NEXT/1 monomethyl NEXT/1 ether:ti,ab,kw OR alpha-zearalanol:ti,ab,kw OR alpha-zearalenol:ti,ab,kw OR altenuene:ti,ab,kw OR beauvericin:ti,ab,kw OR beta-cyclopiazonic NEXT/1 acid:ti,ab,kw OR beta-zearalanol:ti,ab,kw OR beta-zearalenol:ti,ab,kw OR citrinin:ti,ab,kw OR cyclochlorotine:ti,ab,kw OR deoxynivalenol:ti,ab,kw OR vomitoxin:ti,ab,kw OR diacetoxyscirpenol:ti,ab,kw OR dihydro-O-methylsterigmatocystin:ti,ab,kw OR dihydroxy-aflavinine:ti,ab,kw OR dihydroxyl-O-methylsterigmatocystin:ti,ab,kw OR ditryptophenaline:ti,ab,kw OR enniatin*:ti,ab,kw OR ergot NEXT/1 alkaloid*:ti,ab,kw OR flavacol:ti,ab,kw OR fumonisin*:ti,ab,kw OR fusarenon:ti,ab,kw OR fusaric NEXT/1 acid:ti,ab,kw OR fusarin NEXT/1 C:ti,ab,kw OR HT NEXT/1 2 NEXT/1 toxin*:ti,ab,kw OR HT2 NEXT/1 toxin*:ti,ab,kw OR hydroxyneoaspergillic NEXT/1 acid:ti,ab,kw OR leporin NEXT/1 C:ti,ab,kw OR luteoskyrin:ti,ab,kw OR methylcitreo-isocoumarin:ti,ab,kw OR moniliformin:ti,ab,kw OR mycotoxin*:ti,ab,kw OR neosolaniol:ti,ab,kw OR nivalenol:ti,ab,kw OR ochratoxin*:ti,ab,kw OR O-methylsterigmatocystin:ti,ab,kw OR paspalinine:ti,ab,kw OR pathotoxin:ti,ab,kw OR patulin:ti,ab,kw OR phomopsin:ti,ab,kw OR penicillic NEXT/1 acid:ti,ab,kw OR roquefortine-C:ti,ab,kw OR rugulosin:ti,ab,kw OR speradine NEXT/1 A:ti,ab,kw OR sterigmatocystin:ti,ab,kw OR T2 NEXT/1 toxin*:ti,ab,kw OR T NEXT/1 2 NEXT/1 toxin*:ti,ab,kw OR NX NEXT/1 toxin*:ti,ab,kw OR tentoxin:ti,ab,kw OR tenuazonic NEXT/1 acid:ti,ab,kw OR trichothecene*:ti,ab,kw OR verrucarin:ti,ab,kw OR versiconol:ti,ab,kw OR versiconol NEXT/1 hemiacetal NEXT/1 acid:ti,ab,kw OR zearalenone:ti,ab,kw OR zearalanone:ti,ab,kw OR mycotoxin*:ti,ab,kw OR fungal NEXT/1 toxin*:ti,ab,kw) **AND** (systematic NEXT/1 review:ti,ab,kw OR meta-analysis:ti,ab,kw OR evidence NEXT/1 map:ti,ab,kw OR systematic NEXT/1 search:ti,ab,kw)  Secondary search string:  NOT (animal not human) |

**Table S4.** Detailed electronic search string for the scoping exercise in registry databases.

| **Electronic search string – Prospero – 44 results – no filters** |
| --- |
| acetyldeoxynivalenol OR aflatoxin OR aspertoxin OR aspergillic acid OR cyclopiazonic acid OR altertoxin OR alternariol OR alternariol monomethyl ether OR zearalanol OR zearalenol OR altenuene OR beauvericin OR cyclopiazonic acid OR citrinin OR cyclochlorotine OR deoxynivalenol OR vomitoxin OR diacetoxyscirpenol OR sterigmatocystin OR aflavinine OR ditryptophenaline OR enniatin* OR ergot alkaloid* OR flavacol OR fumonisin* OR fusarenon OR fusaric acid OR fusarin C OR HT 2 toxin OR HT2 toxin OR hydroxyneoaspergillic acid OR leporin C OR luteoskyrin OR methylcitreo-isocoumarin OR moniliformin OR mycotoxin* OR neosolaniol OR nivalenol OR ochratoxin* OR paspalinine OR pathotoxin OR patulin OR phomopsin OR penicillic acid OR roquefortine OR rugulosin OR speradine A OR T2 toxin* OR T 2 toxin* OR NX toxin* OR tentoxin OR tenuazonic acid OR trichothecene* OR verrucarin OR versiconol OR versiconol hemiacetal acid OR zearalenone OR zearalanone OR fungal toxin |
| **Electronic search string – OSF Registry – 14 results – no filters** |
| (acetyldeoxynivalenol OR aflatoxin OR aspertoxin OR altertoxin OR alternariol OR zearalanol OR zearalenol OR altenuene OR beauvericin OR citrinin OR cyclochlorotine OR deoxynivalenol OR vomitoxin OR diacetoxyscirpenol OR sterigmatocystin OR aflavinine OR ditryptophenaline OR enniatin* OR “ergot alkaloid*” OR flavacol OR fumonisin* OR fusarenon OR fusarin OR leporin OR luteoskyrin OR methylcitreo-isocoumarin OR moniliformin OR mycotoxin* OR neosolaniol OR nivalenol OR ochratoxin* OR paspalinine OR pathotoxin OR patulin OR phomopsin OR roquefortine OR rugulosin OR speradine OR tentoxin OR tenuazonic OR trichothecene* OR verrucarin OR versiconol OR zearalenone OR zearalanone) AND (“systematic review” OR meta-analysis OR “evidence map” OR “systematic search”) |
| **Electronic search string – Zenodo – 16 results – filter: only publications + keywords: mycotoxin, mycotoxins** |
| mycotoxin* OR aflatoxin* OR enniatin* OR zearalenone OR deoxynivalenol OR ochratoxin |

**Table S5A.** Author(s), title, PECO elements and core methodology of the 22 review articles found and deemed relevant during scoping.

|  | **Author(s)** | **Title** | **Population** | **Exposure** | **Control** | **Outcome** | **Well-articulated PECO statement** | **transparent and valid literature searches** | **transparent and valid risk of bias assessment** | **transparent and valid certainty/confidence assessment** |
| --- | --- | --- | --- | --- | --- | --- | --- | --- | --- | --- |
| ***Embase*** | | | | | | | | | | |
| *412 out of scope* | Alvito P. et al. 2022 | Mycotoxin Exposure during the First 1000 Days of Life and Its Impact on Children’s Health: A Clinical Overview | Subpopulation: Pregnant women and children up to 2 years of age | Multiple | N.R. | Multiple - adverse pregnancy outcomes and child health | No | No | No | No |
|  | Polak-Śliwińska M. et al. 2021 | Trichothecenes in Food and Feed, Relevance to Human and Animal Health and Methods of Detection: A Systematic Review | General population | Single (trichothecenes) | N.R. | Multiple – immunotoxicity & gastrointestinal symptology | No | No | No | No |
|  | Gönenç İ.M. et al. 2020 | Mycotoxin exposure and pregnancy | Subpopulation: pregnant women | Multiple | N.R. | Multiple - adverse pregnancy outcomes | No | No | No | No |
|  | Claeys L. et al. 2020 | Mycotoxin exposure and human cancer risk: A systematic review of epidemiological studies | General population | Multiple | N.R. | Single - cancer | No | Yes^A^ | No | Yes^C^ |
|  | Mekuria A.N. et al. 2020 | Aflatoxins as a risk factor for liver cirrhosis: A systematic review and meta-analysis | General population | Single (aflatoxins) | N.R. | Single - liver cirrhosis | No | No | Yes | Yes^D^ |
|  | Kyei N.N.A. et al. 2020 | Maternal mycotoxin exposure and adverse pregnancy outcomes: a systematic review | Subpopulation: pregnant women | Multiple | No detection or lower levels | Multiple - adverse pregnancy outcomes | Yes | Yes^A^ | Yes^C^ | Yes^C^ |
|  | Tesfamariam K. et al. 2020 | Dietary mycotoxins exposure and child growth, immune system, morbidity, and mortality: a systematic literature review | Subpopulation: pregnant & nursing women, children up to years of age | Multiple | N.R. | Multiple - adverse pregnancy outcomes and child health | No | Yes^A^ | Yes^C,E^ | Yes^C,F^ |
|  | Githang'a D. et al. 2019 | The effects of exposures to mycotoxins on immunity in children: A systematic review | Subpopulation: children up to 12 years of age | Multiple | N.R. | Single - immunity | No | Yes^A^ | Yes^G^ | Yes^G^ |
|  | Mupunga I. et al. 2017 | Peanuts, aflatoxins and undernutrition in children in Sub-Saharan Africa | Subpopulation: children | Single - aflatoxins | N.R. | Single - malnutrition | No | No | No | No |
|  | Smith L.E. et al. 2017 | Aflatoxin exposure during pregnancy, maternal anemia, and adverse birth outcomes | Subpopulation: pregnant women | Single - aflatoxins | N.R. | Multiple - adverse pregnancy outcomes | No | No | No | No |
|  | Li D. et al. 2016 | The effects of T-2 toxin on the prevalence and development of Kashin-Beck disease in China: A meta-analysis and systematic review | General population | Single - T-2 toxin | N.R. | Single - Kashin-Beck disease | No | Yes^A,B^ | No | Yes^H^ |
|  | Bui-Klimke T.R. et al. 2015 | Ochratoxin A and human health risk: a review of the evidence | General population | Single - ochratoxin | N.R. | Single - renal disease | No | Yes^A,B^ | No | No |
|  | Liu Y. et al. 2012 | Population attributable risk of aflatoxin-related liver cancer: Systematic review and meta-analysis | General population | Single - aflatoxins | N.R. | Single - liver cancer | No | Yes^A,B^ | No | No |
| ***Pubmed*** | | | | | | | | | | |
| *291 out of scope, 8 duplicates with Embase* | Kinkade C.W. et al. 2021 | Impact of fusarium-derived mycoestrogens on female reproduction: a systematic review. | General population (& animals) | Single - zearalenone and metabolites | N.R. | Multiple - female reproduction | Yes | Yes | Yes^I^ | No |
| ***Prospero*** | | | | | | | | | | |
| *36 out of scope* | Abdurezak U. et al. 2021 | Mycotoxin and adverse pregnancy outcomes: A systematic review and meta-analysis | Subpopulation: pregnant women | Multiple | No detection or lower levels | Multiple - adverse pregnancy outcomes | Yes | Yes | Yes^D^ | Yes^C^ |
|  | Githang'a D. et al. 2019 | The effects of mycotoxins on the immune status in children | published in Embase | | | | | | | |
|  | Seetha A. et al. 2021 | Establishing the link between dietary mycotoxin exposure and micronutrient deficiency, liver disorders and the effect of interventions on exposure mitigation: a systematic review and meta-analysis | General population | Single - aflatoxins | Non-exposed, crops without mitigation technology | Multiple - liver disorders, nutritional status | Yes | Yes^B^ | Yes^E^ | No |
|  | Tesfamariam K. et al. 2020 | Dietary mycotoxins exposure and child growth, immune system, morbidity, and mortality: a systematic literature review | published in Embase | | | | | | | |
|  | Abrham A. et al. 2019 | Effect of aflatoxin B1 exposure on the incidence of hepatocellular carcinoma with and without HBV, HCV, liver cirrhosis and alcohol consumption: systematic review and meta-analysis | General population | Single - AFB1 | Hepatocellular carcinoma patients with undetectable/low Aflatoxin B1 in Serum and/or Urine. | Single - liver cancer | Yes | No | Yes^J^ | Yes^C^ |
|  | Mekuria A.N. et al. 2020 | Aflatoxins as a risk factor for liver cirrhosis: a systematic review and meta-analysis | published in Embase | |  |  |  |  |  |  |
|  | Motbaynor et al. 2019 | Aflatoxin and growth impairment in children: A systematic review and meta-analysis | Subpopulation: children | Single - aflatoxins | Children who are not taking aflatoxin by food | Single - child growth impairement | Yes | No | Yes^D^ | No |
|  | Kinkade C.W. et al. 2021 | Zearalenone and reproduction: a systematic review of the impact of ZEA exposure in humans as well as in vitro and in vivo model systems | published in Embase | |  |  |  |  |  |  |

Note: all articles retrieved from Cochrane Library, OSF Registry and Zenodo were out of scope of the current review. N.R. = not reported. ^A^ = no scoping performed, ^B^ = no Mesh or Emtree terms included, ^C^ = Newscatle-Ottawa scale, ^D^ = Joanna Briggs Institute critical appraisal checklist for case-control and cohort studies, ^E^ = Cochrane methodology, ^F^ = GRADE, ^G^ = Joanna Briggs Institute tool for appraisal of systematic reviews for prevalence studies, ^H^ = Agency for Healthcare Research and Quality standard, ^I^ = ToxR tool, ^J^ = only publication bias assessed via visual inspection funnel plot.

**Table S5B.** Objective(s) and key conclusion of the 22 review articles withheld during scoping.

| **Authors** | **Objective** | **Key conclusion** |
| --- | --- | --- |
| Alvito P. et al. 2022 | The aim of this narrative review was to gather data from the literature on internal and external exposure to mycotoxins and their short- and long-term health risks during intrauterine life and early childhood for vulnerable populations, such as pregnant women and young children, in different continents. | Maternal internal exposure to aflatoxins is associated with fetal growth restriction, while exposure to fumonisins increases the risk of offspring’s neural tube defects. Both external and internal exposure to aflatoxins and fumonisins in children are reported to be associated with growth impairment. |
| Polak-Śliwińska M. et al. 2021 | the current article aims to update a systematic review on the occurrence of trichothecenes in 2011–2020 and their toxic effects and to provide a summary of the discussions on the potential public health concerns specifically related to mycotoxin residues in foods associated with the exposure of farm animals to mycotoxin-contaminated feeds. Moreover, the article discusses the methods of their detection. | This review shows the occurrence of trichothecenes in food and feed in 2011–2020 and their toxic effects and provides a summary of the discussions on the potential public health concerns specifically related to trichothecenes residues in foods associated with the exposure of farm animals to mycotoxin-contaminated feeds and impact to human health. |
| Gönenç İ.M. et al. 2020 | This review aims to investigate the adverse effects of mycotoxins during pregnancy. | There are some evidences that mycotoxins can lead to negative pregnancy outcomes. These possible negative effects have been determined to be lower birth weight, neonatal jaundice, fetal loss, fetal defects, preterm birth, maternal anemia, and preeclampsia. However, more evidence is needed on this topic. |
| Claeys L. et al. 2020 | Mycotoxin exposure and human cancer risk: A systematic review of epidemiological studies | Overall, a positive association between the consumption of aflatoxin-contaminated foods and primary liver cancer risk was verified. Two case–control studies in Africa investigated the relationship between zearalenone and its metabolites and breast cancer risk, though conflicting results were reported. Two case–control studies investigated the association between hepatocellular carcinoma and fumonisin B1 exposure, but no significant associations were observed. |
| Mekuria A.N. et al. 2020 | This study aimed to assess the association between aflatoxin exposure and the risk of liver cirrhosis. | The present meta-analysis suggests that aflatoxin exposure is associated with a higher risk of liver cirrhosis. |
| Kyei N.N.A. et al. 2020 | This study aimed to synthesize the evidence from epidemiological studies on the relationship between maternal or fetal exposure to different mycotoxins and the occurrence of adverse pregnancy outcomes. | There is already some evidence to suggest that exposure to mycotoxins during pregnancy may have detrimental effects on pregnancy outcomes. |
| Tesfamariam K. et al. 2020 | The aim of this study was to systematically review associations between dietary mycotoxins exposure and child growth and morbidity of children aged 5 years or younger. | Uncertainty remains whether mycotoxins exposure affects child growth, immunity and mortality and the overall quality of the evidence is very low. Overall however, we cannot rule out a possible association between dietary mycotoxins, in particular, AF and FUM and child malnutrition. |
| Githang'a D. et al. 2019 | This review aims to describe the effects of mycotoxin exposure on the immunity in children to explore if any gaps exists that may be contributing to the sub-optimal vaccine effectiveness alluded to in the introduction. | Exposure to mycotoxins was positively associated with low birth weight and concentration of antibodies to asexual malaria parasites and hepatitis B surface antigen, and negatively associated with death and sIgA, antibodies to pneumococcal antigen 23. |
| Mupunga I. et al. 2017 | This article reviews the nutritional value and aflatoxin contamination of peanuts, the role they play in the development of childhood malnutrition (including the different theories of aetiology) and immunological problems in children. | Aflatoxins contribute to malnutrition by interfering with intestinal integrity and hepatic metabolism. |
| Smith L.E. et al. 2017 | In this article, we aimed to conduct a narrative review to evaluate the evidence for a potential role of aflatoxin exposure and anemia in intrauterine growth retardation, preterm birth, and pregnancy loss. | Findings suggest that aflatoxin exposure during pregnancy may impair fetal growth. Only one human study investigated aflatoxin exposure and prematurity, and no studies investigated its relationship with pregnancy loss, but animal studies suggest aflatoxin exposure may increase risk for prematurity and pregnancy loss. |
| Li D. et al. 2016 | To reveal the influence of T-2 toxin detection rate and detection amount in food samples on Kashin–Beck disease (KBD), and define a linking mechanism between T-2 toxin induced chondrocytes or cartilage damage and KBD pathological changes, seven electronic databases were searched to obtain epidemiological and experimental studies. | The amount of T-2 toxin detected has a more significant influence on KBD prevalence and development as compared to the T-2 toxin detection rate. Besides, the T-2 toxin induces chondrocyte and cartilage damage through apoptosis, catabolism promotion and intracellular impairment, which is similar to the KBD change. |
| Bui-Klimke T.R. et al. 2015 | This study aims to review the epidemiological literature on OTA exposure and adverse health effects in different populations worldwide, and assesses the potential human health risks of OTA exposure. | With one exception, there appears to be no statistically significant evidence for human health risks associated with OTA exposure. |
| Liu Y. et al. 2012 | In our systematic review and meta-analysis of epidemiological studies, summary odds ratios (ORs) of aflatoxin-related HCC with 95% confidence intervals were calculated in HBV+ and HBV individuals, as well as the general population. We calculated the PAR of aflatoxin-related HCC for each study as well as the combined studies, accounting for HBV status. | In high exposure areas, aflatoxin multiplicatively interacts with HBV to induce, HCC; reducing aflatoxin exposure to non-detectable levels could reduce HCC cases in high-risk areas by about 23%. The decreasing PAR of aflatoxin-related HCC reflects the benefits of public health interventions to reduce aflatoxin and HBV. |
| Kinkade C.W. et al. 2021 | The objective of this systematic review was to summarize the in vitro, in vivo, and epidemiological literature and evaluate the potential impact of ZEN, zeranol, and their metabolites (commonly referred to as mycoestrogens) on female reproductive outcomes. | In non-pregnant animals, mycoestrogens alter follicular profiles in the ovary, disrupt estrus cycling, and increase myometrium thickness. Furthermore, during pregnancy, mycoestrogen exposure contributes to placental hemorrhage, stillbirth, and impaired fetal growth. No epidemiological studies fitting the inclusion criteria were identified. |
| Abdurezak U. et al. 2021 | To asses if there is a link between maternal mycotoxin exposure and adverse pregnancy outcomes such as (premature birth, low birth weight, small for gestational age and still birth). | Review ongoing. |
| Seetha A. et al. 2021 | To assess if aflatoxin exposure leads to liver disorders and reduced nutritional status. | Review ongoing. |
| Abrham A. et al. 2019 | To assess what the effect is of Aflatoxin B1 exposure on the incidence of Hepatocellular Carcinoma and if there is any synergism between Aflatoxin B1 exposure, Hepatitis B Virus (HBV), and Hepatitis C Virus (HCV) infection, Liver Cihirosis and Alcohol consumption for incidence of Hepatocellular Carcinoma. | Review ongoing. |
| Motbaynor et al. 2019 | To assess if there is a relationship between aflatoxin and child growth impairement. | Review ongoing. |

**Table S6.** Members of the review team who have been assigned to core SR competencies.

| **Assigned role** | **Author** |
| --- | --- |
| Information science | TG, YB, MDB |
| Evidence Appraisal | TG, MDB, CL |
| Statistical methods | NNT, TMM, KT, CL |
| Topic expertise |  |
| Mycotoxins | TG, TMM, FV, YB, SDS, MDB |
| Human health | TMM, KT, FV, CL, MDB |
| Nutrition epidemiology | TMM, KT, CL, MDB |
| Systematic review methods | TG, TMM, KT, CL, MDB, SDS* |
| Guarantor of review | TG, CL, SDS, MDB |

Note: * = Healthcare Knowledge Center Ghent (KCGG, <https://www.kcgg.ugent.be/>) was consulted on the development of the structure of the literature search string.

**Table S7.** Mycotoxins classified according to the IARC Monograph that identifies environmental causes of cancer in humans.

| **IARC classification** | **Mycotoxin** |
| --- | --- |
| Group 1: the agent is carcinogenic to humans | AFB1, AFB2, AFG1, AFG2, AFM1 |
| Group 2A: the agent is probably carcinogenic to humans | - |
| Group 2B: the agent is possibly carcinogenic to humans | OTA, FB1, FB2, STC, Fusarin C |
| Group 3: the agent is not classifiable as to its carcinogenicity to humans | DON, ZEN, Fusarenone X, CIT, PAT |
| Group 4: the agent is probably not carcinogenic to humans | - |

Note: AFB1 = aflatoxin B1; AFB2 = aflatoxin B2; AFG1 = aflatoxin G1; AFG2 = aflatoxin G2; AFM1 = aflatoxin M1; CIT = citrinin; DON = deoxynivalenol; FB1 = fumonisin B1; FB2 = fumonisin B2; OTA = ochratoxin A; PAT = patulin; STC = sterigmatocystin; ZEN = zearalenone.

**Table S8.** Detailed electronic search string for PubMed.

| **Health risk category** | **Electronic search string – no filters** |
| --- | --- |
| Cancer | Primary search string:  (3-acetyldeoxynivalenol[tiab] OR 15-acetyldeoxynivalenol[tiab] OR afla*[tiab] OR aspertoxin[tiab] OR “aspergillic acid”[tiab] OR “cyclopiazonic acid”[tiab] OR altertoxin[tiab] OR alternariol[tiab] OR “alternariol monomethyl ether”[tiab] OR alpha-zearalanol[tiab] OR alpha-zearalenol[tiab] OR altenuene[tiab] OR beauvericin[tiab] OR beta-cyclopiazonic acid[tiab] OR beta-zearalanol[tiab] OR beta-zearalenol[tiab] OR citrinin[tiab] OR cyclochlorotine[tiab] OR deoxynivalenol[tiab] OR vomitoxin[tiab] OR diacetoxyscirpenol[tiab] OR dihydro-O-methylsterigmatocystin[tiab] OR dihydroxy-aflavinine[tiab] OR dihydroxyl-O-methylsterigmatocystin[tiab] OR ditryptophenaline[tiab] OR enniatin*[tiab] OR “ergot alkaloid*”[tiab] OR flavacol[tiab] OR fumonisin*[tiab] OR “fusarenon X”[tiab] OR “fusaric acid”[tiab] OR “fusarin C”[tiab] OR “HT2 toxin”[tiab] OR “HT 2 toxin”[tiab] OR “hydroxyneoaspergillic acid”[tiab] OR “leporin C”[tiab] OR luteoskyrin[tiab] OR methylcitreo-isocoumarin[tiab] OR moniliformin[tiab] OR mycotoxin*[tiab] OR neosolaniol[tiab] OR nivalenol[tiab] OR ochratoxin*[tiab] OR O-methylsterigmatocystin[tiab] OR paspalinine[tiab] OR pathotoxin[tiab] OR patulin[tiab] OR phomopsin[tiab] OR “penicillic acid”[tiab] OR “roquefortine C”[tiab] OR rugulosin[tiab] OR speradine A[tiab] OR sterigmatocystin[tiab] OR “T2 toxin”[tiab] OR “T 2 toxin” OR “NX toxin”[tiab] OR tentoxin[tiab] OR “tenuazonic acid”[tiab] OR trichothecene*[tiab] OR verrucarin[tiab] OR versiconol[tiab] OR zearalenone[tiab] OR zearalanol[tiab] OR zearalenol[tiab] OR mycotoxin*[tiab] OR “fungal toxin*”[tiab] OR “mycotoxins”[MeSH Terms] OR “aflatoxins”[MeSH Terms] OR “ergot alkaloids”[MeSH Terms] OR “fusaric acid”[MeSH Terms] OR “alternariol”[Supplementary Concept] OR “alternariol monomethyl ether”[Supplementary Concept] OR “deoxynivalenol”[Supplementary Concept] OR “altertoxin”[Supplementary Concept] OR “fusarin C”[Supplementary Concept] OR “enniatins”[Supplementary Concept] OR “moniliformin”[Supplementary Concept] OR “versiconol”[Supplementary Concept] OR “zearalenol”[Supplementary Concept]) **AND** ("Neoplasms"[Mesh] OR neoplas*[tiab] OR paraneoplas*[tiab] OR tumor*[tiab] OR tumour*[tiab] OR cancer*[tiab] OR carcinogen*[tiab] OR precancerous[tiab] OR teratoma*[tiab] OR malignan*[tiab] OR oncolog*[tiab] OR oncogene*[tiab] OR carcinom*[tiab] OR sarcom*[tiab] OR carcinosarcom*[tiab] adenocarcinom*[tiab] OR adenosarcom*[tiab] OR adenom*[tiab] OR melanom*[tiab] OR gonadoblastom*[tiab] OR hepatoblastom*[tiab] OR blastoma*[tiab] OR chordoma*[tiab] OR germinoma*[tiab] OR leukemia*[tiab] OR lymphoma*[tiab] OR lymphangioma*[tiab] OR lymphangiosarcoma*[tiab] OR meningioma*[tiab] OR mesenchymoma*[tiab] OR mesonephroma*[tiab] OR plasmacytoma*[tiab] OR teratocarcinoma*[tiab] OR hodgkin[tiab] OR non-hodgkin[tiab] OR myeloma*[tiab] OR kahler[tiab] OR metastas*[tiab])  Secondary search string:  NOT (“Animals”[mesh] NOT “Humans”[mesh]) |
| Non-carcinogenic diseases | Primary search string:  (3-acetyldeoxynivalenol[tiab] OR 15-acetyldeoxynivalenol[tiab] OR afla*[tiab] OR aspertoxin [tiab] OR “aspergillic acid”[tiab] OR “cyclopiazonic acid”[tiab] OR altertoxin[tiab] OR alternariol[tiab] OR “alternariol monomethyl ether”[tiab] OR alpha-zearalanol[tiab] OR alpha-zearalenol[tiab] OR altenuene[tiab] OR beauvericin[tiab] OR beta-cyclopiazonic acid[tiab] OR beta-zearalanol[tiab] OR beta-zearalenol[tiab] OR citrinin[tiab] OR cyclochlorotine[tiab] OR deoxynivalenol[tiab] OR vomitoxin[tiab] OR diacetoxyscirpenol[tiab] OR dihydro-O-methylsterigmatocystin[tiab] OR dihydroxy-aflavinine[tiab] OR dihydroxyl-O-methylsterigmatocystin[tiab] OR ditryptophenaline[tiab] OR enniatin*[tiab] OR “ergot alkaloid*”[tiab] OR flavacol[tiab] OR fumonisin*[tiab] OR “fusarenon X”[tiab] OR “fusaric acid”[tiab] OR “fusarin C”[tiab] OR “HT2 toxin”[tiab] OR “HT 2 toxin”[tiab] OR “hydroxyneoaspergillic acid”[tiab] OR “leporin C”[tiab] OR luteoskyrin[tiab] OR methylcitreo-isocoumarin[tiab] OR moniliformin[tiab] OR mycotoxin*[tiab] OR neosolaniol[tiab] OR nivalenol[tiab] OR ochratoxin*[tiab] OR O-methylsterigmatocystin[tiab] OR paspalinine[tiab] OR pathotoxin[tiab] OR patulin[tiab] OR phomopsin[tiab] OR “penicillic acid”[tiab] OR “roquefortine C”[tiab] OR rugulosin[tiab] OR speradine A[tiab] OR sterigmatocystin[tiab] OR “T2 toxin”[tiab] OR “T 2 toxin” OR “NX toxin”[tiab] OR tentoxin[tiab] OR “tenuazonic acid”[tiab] OR trichothecene*[tiab] OR verrucarin[tiab] OR versiconol[tiab] OR zearalenone[tiab] OR zearalanol[tiab] OR zearalenol[tiab] OR mycotoxin*[tiab] OR “fungal toxin*”[tiab] OR “mycotoxins”[MeSH Terms] OR “aflatoxins”[MeSH Terms] OR “ergot alkaloids”[MeSH Terms] OR “fusaric acid”[MeSH Terms] OR “alternariol”[Supplementary Concept] OR “alternariol monomethyl ether”[Supplementary Concept] OR “deoxynivalenol”[Supplementary Concept] OR “altertoxin”[Supplementary Concept] OR “fusarin C”[Supplementary Concept] OR “enniatins”[Supplementary Concept] OR “moniliformin”[Supplementary Concept] OR “versiconol”[Supplementary Concept] OR “zearalenol”[Supplementary Concept]) **AND** (“Central Nervous System Diseases”[Mesh] OR “central nervous system disease*”[tiab] OR encephalopath*[tiab] OR hepatoencephalopath*[tiab] OR neurotoxi*[tiab] OR neuropath*[tiab] OR neurodegenerati*[tiab] OR "Parkinsonian Disorders"[Mesh] OR parkinson*[tiab] OR "Alzheimer Disease"[Mesh] OR alzheimer*[tiab] OR “senile dementia*”[tiab] OR neurobehavio*[tiab] OR “nodding syndrome”[tiab] OR tremor*[tiab] OR “brain”[tiab] OR “gastrointestinal diseases”[Mesh] OR “gastrointestinal disease*”[tiab] OR “gastrointestinal toxic*”[tiab] OR enteropath*[tiab] OR "Inflammatory Bowel Diseases"[Mesh] OR “inflammatory bowel dis*”[tiab] OR crohn*[tiab] OR ileocolitis[tiab] OR ileitis[tiab] OR ibd[tiab] OR colitis[tiab] OR enteritis[tiab] OR gastrointestin*[tiab] OR intestine*[tiab] OR bowel[tiab] OR colon[tiab] OR "Kidney Diseases"[Mesh] OR "Kidney Function Tests"[Mesh] OR nephrot*[tiab] OR anuria[tiab] OR “diabetes insipidus”[tiab] OR nephriti*[tiab] OR pyelitis[tiab] OR perinephriti*[tiab] OR glycosuria[tiab] OR uremia[tiab] OR polyuri*[tiab] OR oliguri*[tiab] OR nephropath*[tiab] OR kidney[tiab] OR renal[tiab] OR hepat*[tiab] OR ˆ“liver diseases”[Mesh] OR cirrhosis[tiab] OR liver*[tiab] OR “esophageal diseases”[Mesh] OR “esophageal disease*”[tiab] OR esophagitis[tiab] OR esophagus[tiab] OR “hematologic diseases”[Mesh] OR “lymphatic diseases”[Mesh] OR “hematologic disease*”[tiab] OR anemia[tiab] OR pancytopenia[tiab] OR leukopenia[tiab] OR “alimentary toxic aleukia”[tiab] OR leukocytosis[tiab] OR “lymphatic disease*”[tiab] OR blood[tiab] OR “lymphatic system”[tiab] OR “immune system”[tiab] OR immunomodulation[tiab] OR “immunological modulation”[tiab] OR immunosuppression[tiab] OR “immunological suppression”[tiab] OR "Thyroid Diseases"[Mesh] OR thyroid[tiab] OR hyperthyroid*[tiab] OR hypothyroid*[tiab] OR goiter*[tiab] OR graves*[tiab] OR hyperthyroxinemi*[tiab] OR hypothyroxinemi*[tiab] OR thyroxinemi*[tiab] OR Thyrotoxi*[tiab] OR myxedema*[tiab] OR myxoedema*[tiab] OR hashimoto*[tiab] OR thyroid[tiab] OR “endocrine system diseases”[Mesh] OR diabetes*[tiab] OR diabetic*[tiab] OR “metabolic syndrome”[Mesh] OR “metabolic syndrome”[tiab] OR “glucose intolerance”[Mesh] OR “hyperglycemia”[Mesh] OR “insulin resistance”[Mesh] OR “glucose intoleran*”[tiab] OR hyperglycemi*[tiab] OR “insulin resistan*”[tiab] OR “endocrine system”[tiab] OR "Cardiovascular Diseases"[Mesh] OR cardiovascul*[tiab] OR heart[tiab]OR cardiomyo*[tiab] OR "Metagenome"[Mesh] OR "Microbiota"[Mesh] OR metagenom*[tiab] OR microbiom*[tiab] OR microbiot*[tiab] OR symbios*[tiab] OR dysbios*[tiab] OR pathosymbio*[tiab] OR flora*[tiab] OR microflora*[tiab] OR infections[Mesh] OR infection*[tiab] OR “chemically-induced disorders”[Mesh] OR “disorders of environmental origin”[Mesh] OR “musculoskeletal diseases”[Mesh] OR “nutritional and metabolic diseases”[Mesh])  Secondary search string:  NOT (“Animals”[mesh] NOT “Humans”[mesh]) |
| Reproductive & developmental conditions | Primary search string:  (3-acetyldeoxynivalenol[tiab] OR 15-acetyldeoxynivalenol[tiab] OR afla*[tiab] OR aspertoxin[tiab] OR “aspergillic acid”[tiab] OR “cyclopiazonic acid”[tiab] OR altertoxin[tiab] OR alternariol[tiab] OR “alternariol monomethyl ether”[tiab] OR alpha-zearalanol[tiab] OR alpha-zearalenol[tiab] OR altenuene[tiab] OR beauvericin[tiab] OR beta-cyclopiazonic acid[tiab] OR beta-zearalanol[tiab] OR beta-zearalenol[tiab] OR citrinin[tiab] OR cyclochlorotine[tiab] OR deoxynivalenol[tiab] OR vomitoxin[tiab] OR diacetoxyscirpenol[tiab] OR dihydro-O-methylsterigmatocystin[tiab] OR dihydroxy-aflavinine[tiab] OR dihydroxyl-O-methylsterigmatocystin[tiab] OR ditryptophenaline[tiab] OR enniatin*[tiab] OR “ergot alkaloid*”[tiab] OR flavacol[tiab] OR fumonisin*[tiab] OR “fusarenon X”[tiab] OR “fusaric acid”[tiab] OR “fusarin C”[tiab] OR “HT2 toxin”[tiab] OR “HT 2 toxin”[tiab] OR “hydroxyneoaspergillic acid”[tiab] OR “leporin C”[tiab] OR luteoskyrin[tiab] OR methylcitreo-isocoumarin[tiab] OR moniliformin[tiab] OR mycotoxin*[tiab] OR neosolaniol[tiab] OR nivalenol[tiab] OR ochratoxin*[tiab] OR O-methylsterigmatocystin[tiab] OR paspalinine[tiab] OR pathotoxin[tiab] OR patulin[tiab] OR phomopsin[tiab] OR “penicillic acid”[tiab] OR “roquefortine C”[tiab] OR rugulosin[tiab] OR speradine A[tiab] OR sterigmatocystin[tiab] OR “T2 toxin”[tiab] OR “T 2 toxin” OR “NX toxin”[tiab] OR tentoxin[tiab] OR “tenuazonic acid”[tiab] OR trichothecene*[tiab] OR verrucarin[tiab] OR versiconol[tiab] OR zearalenone[tiab] OR zearalanol[tiab] OR zearalenol[tiab] OR mycotoxin*[tiab] OR “fungal toxin*”[tiab] OR “mycotoxins”[MeSH Terms] OR “aflatoxins”[MeSH Terms] OR “ergot alkaloids”[MeSH Terms] OR “fusaric acid”[MeSH Terms] OR “alternariol”[Supplementary Concept] OR “alternariol monomethyl ether”[Supplementary Concept] OR “deoxynivalenol”[Supplementary Concept] OR “altertoxin”[Supplementary Concept] OR “fusarin C”[Supplementary Concept] OR “enniatins”[Supplementary Concept] OR “moniliformin”[Supplementary Concept] OR “versiconol”[Supplementary Concept] OR “zearalenol”[Supplementary Concept]) **AND** (((Child*[tiab] OR Newborn[tiab] OR newborns[tiab] OR neonate[tiab] OR neonates[tiab] OR neonatal[tiab] OR infant[mh] OR infant[tiab] OR infants[tiab] OR “child, preschool”[mh] OR preschool[tiab] OR “pre school”[tiab] OR pre-school[tiab] OR toddler[tiab] OR toddlers[tiab] OR pediatric*[tiab] OR paediatric[tiab] OR “young children”[tiab] OR “under five years”[tiab] OR “under 5 years”[tiab] OR utero[tiab] OR foetal[tiab] OR fetal[tiab]) AND (growth[tiab] OR stunting[tiab] OR stunted[tiab] OR wasted[tiab] OR wasting[tiab] OR underweight[tiab] OR “short stature”[tiab] OR “malnutrition”[mh] OR malnutrition[tiab] OR malnourished[tiab] OR “mid upper arm circumference”[tiab] OR “mid-upper arm circumference”[tiab] OR MUAC[tiab] OR “linear growth”[tiab] OR “growth faltering”[tiab] OR “childhood stunting”[tiab] OR “growth impairment”[tiab] OR “growth retardation”[tiab] OR “growth deficit”[tiab] OR ”child growth”[tiab] OR “growth restricted”[tiab] OR birthweight[tiab] OR “birth weight”[tiab] OR “small for gestational age”[tiab] OR “small-for-gestational age”[tiab] OR length-for-age[tiab] OR height-for-age[tiab] OR weight-for-height[tiab] OR weight-for-age[tiab] OR emaciated[tiab] OR thin[tiab] OR “protein-energy malnutrition”[tiab] OR “immune system”[mh] OR “immune system”[tiab] OR “immunity”[mh] OR “immune status”[tiab] OR antibody[tiab] OR enteropathy[tiab] OR immunosuppression[tiab] OR immunodeficiency[tiab] OR immunomodulation[tiab] OR immunoglobulin[tiab] OR immunotoxin[tiab] OR immunocompromising[tiab] OR morbidity[tiab] OR infections OR jaundice[tw] OR hepatitis[tiab] OR outbreak[tiab] OR marasmus[tiab] OR kwashiorkor[tiab] OR “marasmic kwashiorkor”[tiab] OR “child mortality”[mh] OR mortality[tiab] OR death[tiab] OR “postnatal mortality”[tiab] OR “infant mortality"[mh] OR “neonatal mortality”[tiab] OR "perinatal death"[mh] OR “postnatal death”[tiab])) OR “infertility”[mh] OR infertile*[tiab] OR “embryonic development”[mh] OR “embryo* development”[tiab] OR “fetal development”[mh] OR “fetal growth retardation”[mh] OR “fetal development*”[tiab] OR “fetal growth*”[tiab] OR “fetal abnormal*”[tiab] OR “placenta"[mh] OR placenta*[tiab] OR "pregnancy"[mh] OR “pregnant women”[mh] OR pregnan*[tiab] OR mother*[tiab] OR maternal[tiab] OR “adverse pregnancy outcome*”[tiab] OR miscarriage[tiab] OR “pregnancy loss”[tiab] OR "Abortion, Spontaneous"[mh] OR abortion*[tiab] OR "Infant, Premature"[mh] OR “premature birth”[mh] OR “premature birth”[tiab] OR “preterm delivery”[tiab] OR "Congenital Abnormalities"[mh] OR “congenital abnormalit*”[tiab] OR "Fetal Death"[mh] OR fetal death[tiab] OR “stillbirth”[mh] OR stillbirth [tiab] OR “still birth”[tiab] OR “pregnancy outcome”[mh] OR “pregnancy outcome*”[tiab])  Secondary search string:  NOT (“Animals”[mesh] NOT “Humans”[mesh]) |

**Table S8.** Detailed electronic search string for Embase.

| **Health risk category** | **Electronic search string – no filters** |
| --- | --- |
| Cancer | Primary search string:  (3-acetyldeoxynivalenol:ti,ab,kw OR 15-acetyldeoxynivalenol:ti,ab,kw OR afla*:ti,ab,kw OR aspertoxin:ti,ab,kw OR ‘aspergillic acid’:ti,ab,kw OR ‘cyclopiazonic acid’:ti,ab,kw OR altertoxin:ti,ab,kw OR alternariol:ti,ab,kw OR ‘alternariol monomethyl ether’:ti,ab,kw OR alpha-zearalanol:ti,ab,kw OR alpha-zearalenol:ti,ab,kw OR altenuene:ti,ab,kw OR beauvericin:ti,ab,kw OR ‘beta-cyclopiazonic acid’:ti,ab,kw OR beta-zearalanol:ti,ab,kw OR beta-zearalenol:ti,ab,kw OR citrinin:ti,ab,kw OR cyclochlorotine:ti,ab,kw OR deoxynivalenol:ti,ab,kw OR vomitoxin:ti,ab,kw OR diacetoxyscirpenol:ti,ab,kw OR dihydro-O-methylsterigmatocystin:ti,ab,kw OR dihydroxy-aflavinine:ti,ab,kw OR dihydroxyl-O-methylsterigmatocystin:ti,ab,kw OR ditryptophenaline:ti,ab,kw OR enniatin*:ti,ab,kw OR ‘ergot alkaloid*’:ti,ab,kw OR flavacol:ti,ab,kw OR fumonisin*:ti,ab,kw OR fusarenon X:ti,ab,kw OR ‘fusaric acid’:ti,ab,kw OR ‘fusarin C’:ti,ab,kw OR ‘HT 2 toxin*’:ti,ab,kw OR ‘HT2 toxin*’:ti,ab,kw OR ‘hydroxyneoaspergillic acid’:ti,ab,kw OR ‘leporin C’:ti,ab,kw OR luteoskyrin:ti,ab,kw OR methylcitreo-isocoumarin:ti,ab,kw OR moniliformin:ti,ab,kw OR mycotoxin*:ti,ab,kw OR neosolaniol:ti,ab,kw OR nivalenol:ti,ab,kw OR ochratoxin*:ti,ab,kw OR O-methylsterigmatocystin:ti,ab,kw OR paspalinine:ti,ab,kw OR pathotoxin:ti,ab,kw OR patulin:ti,ab,kw OR phomopsin:ti,ab,kw OR ‘penicillic acid’:ti,ab,kw OR roquefortine-C:ti,ab,kw OR rugulosin:ti,ab,kw OR ‘speradine A’:ti,ab,kw OR sterigmatocystin:ti,ab,kw OR ‘T2 toxin*’:ti,ab,kw OR ‘T 2 toxin*’:ti,ab,kw OR ‘NX toxin*’:ti,ab,kw OR tentoxin:ti,ab,kw OR ‘tenuazonic acid’:ti,ab,kw OR trichothecene*:ti,ab,kw OR verrucarin:ti,ab,kw OR versiconol:ti,ab,kw OR ‘versiconol hemiacetal acid’:ti,ab,kw OR zearalenone:ti,ab,kw OR zearalanone:ti,ab,kw OR mycotoxin*:ti,ab,kw OR ‘fungal toxin*’:ti,ab,kw OR ‘mycotoxin’/exp OR ‘aflatoxin’/exp OR ‘aflatoxin B’/exp OR ‘aflatoxin B1’/exp OR ‘aflatoxin B2’/exp OR ‘aflatoxin G1’/exp OR ‘aflatoxin G2’/exp OR ‘aflatoxin M1’/exp OR ‘alpha zearalenol’/exp OR ‘alternariol’/exp OR ‘beta zearalenol’/exp OR ‘diacetoxyscirpenol’/exp OR ‘fumonisin’/exp OR ‘fumonisin A1’/exp OR ‘fumonisin B1’/exp OR ‘fumonisin B2’/exp OR ‘fusarenon X’/exp OR ‘HT 2 toxin’/exp OR ‘luteoskyrin’/exp OR ‘masked mycotoxin’/exp OR ‘moniliformin’/exp OR ‘nivalenol’/exp OR ‘ochratoxin’/exp OR ‘patulin’/exp OR ‘sterigmatocystin’/exp OR ‘T 2 toxin’ OR ‘tenuazonic acid’/exp OR ‘trichothecene’/exp OR ‘verrucarin A’/exp OR ‘verrucarin A derivate’/exp OR ‘versicolorin A’/exp OR ‘vomitoxin’/exp OR ‘zearalenone’/exp OR ‘penicillic acid’/exp OR ‘cyclopiazonic acid’/exp OR ‘beauvericin’/exp OR ‘citrinin’/exp OR “enniatin’/exp OR ‘ergot alkaloid’/exp OR ‘fusaric acid’/exp OR ‘neosolaniol’/exp) **AND** ('neoplasm'/exp OR neoplas*:ti,ab,kw OR paraneoplas*:ti,ab,kw OR tumor*:ti,ab,kw OR tumour*:ti,ab,kw OR cancer*:ti,ab,kw OR carcinogen*:ti,ab,kw OR precancerous:ti,ab,kw OR teratoma*:ti,ab,kw OR malignan*:ti,ab,kw OR oncolog*:ti,ab,kw OR oncogene*:ti,ab,kw OR carcinom*:ti,ab,kw OR sarcom*:ti,ab,kw OR carcinosarcom*:ti,ab,kw OR adenocarcinom*:ti,ab,kw OR adenosarcom*:ti,ab,kw OR adenom*:ti,ab,kw OR melanom*:ti,ab,kw OR gonadoblastom*:ti,ab,kw OR hepatoblastom*:ti,ab,kw OR blastoma*:ti,ab,kw OR chordoma*:ti,ab,kw OR germinoma*:ti,ab,kw OR leukemia*:ti,ab,kw OR lymphoma*:ti,ab,kw OR lymphangioma*:ti,ab,kw OR lymphangiosarcoma*:ti,ab,kw OR meningioma*:ti,ab,kw OR mesenchymoma*:ti,ab,kw OR mesonephroma*:ti,ab,kw OR plasmacytoma*:ti,ab,kw OR teratocarcinoma*:ti,ab,kw OR hodgkin:ti,ab,kw OR non-hodgkin:ti,ab,kw OR myeloma*:ti,ab,kw OR kahler:ti,ab,kw OR metastas*:ti,ab,kw)  Secondary search string:  NOT (‘animal’/exp NOT ‘human’/exp) |
| Non-carcinogenic diseases | Primary search string:  (3-acetyldeoxynivalenol:ti,ab,kw OR 15-acetyldeoxynivalenol:ti,ab,kw OR afla*:ti,ab,kw OR aspertoxin:ti,ab,kw OR ‘aspergillic acid’:ti,ab,kw OR ‘cyclopiazonic acid’:ti,ab,kw OR altertoxin:ti,ab,kw OR alternariol:ti,ab,kw OR ‘alternariol monomethyl ether’:ti,ab,kw OR alpha-zearalanol:ti,ab,kw OR alpha-zearalenol:ti,ab,kw OR altenuene:ti,ab,kw OR beauvericin:ti,ab,kw OR ‘beta-cyclopiazonic acid’:ti,ab,kw OR beta-zearalanol:ti,ab,kw OR beta-zearalenol:ti,ab,kw OR citrinin:ti,ab,kw OR cyclochlorotine:ti,ab,kw OR deoxynivalenol:ti,ab,kw OR vomitoxin:ti,ab,kw OR diacetoxyscirpenol:ti,ab,kw OR dihydro-O-methylsterigmatocystin:ti,ab,kw OR dihydroxy-aflavinine:ti,ab,kw OR dihydroxyl-O-methylsterigmatocystin:ti,ab,kw OR ditryptophenaline:ti,ab,kw OR enniatin*:ti,ab,kw OR ‘ergot alkaloid*’:ti,ab,kw OR flavacol:ti,ab,kw OR fumonisin*:ti,ab,kw OR fusarenon X:ti,ab,kw OR ‘fusaric acid’:ti,ab,kw OR ‘fusarin C’:ti,ab,kw OR ‘HT 2 toxin*’:ti,ab,kw OR ‘HT2 toxin*’:ti,ab,kw OR ‘hydroxyneoaspergillic acid’:ti,ab,kw OR ‘leporin C’:ti,ab,kw OR luteoskyrin:ti,ab,kw OR methylcitreo-isocoumarin:ti,ab,kw OR moniliformin:ti,ab,kw OR mycotoxin*:ti,ab,kw OR neosolaniol:ti,ab,kw OR nivalenol:ti,ab,kw OR ochratoxin*:ti,ab,kw OR O-methylsterigmatocystin:ti,ab,kw OR paspalinine:ti,ab,kw OR pathotoxin:ti,ab,kw OR patulin:ti,ab,kw OR phomopsin:ti,ab,kw OR ‘penicillic acid’:ti,ab,kw OR roquefortine-C:ti,ab,kw OR rugulosin:ti,ab,kw OR ‘speradine A’:ti,ab,kw OR sterigmatocystin:ti,ab,kw OR ‘T2 toxin*’:ti,ab,kw OR ‘T 2 toxin*’:ti,ab,kw OR ‘NX toxin*’:ti,ab,kw OR tentoxin:ti,ab,kw OR ‘tenuazonic acid’:ti,ab,kw OR trichothecene*:ti,ab,kw OR verrucarin:ti,ab,kw OR versiconol:ti,ab,kw OR ‘versiconol hemiacetal acid’:ti,ab,kw OR zearalenone:ti,ab,kw OR zearalanone:ti,ab,kw OR mycotoxin*:ti,ab,kw OR ‘fungal toxin*’:ti,ab,kw OR ‘mycotoxin’/exp OR ‘aflatoxin’/exp OR ‘aflatoxin B’/exp OR ‘aflatoxin B1’/exp OR ‘aflatoxin B2’/exp OR ‘aflatoxin G1’/exp OR ‘aflatoxin G2’/exp OR ‘aflatoxin M1’/exp OR ‘alpha zearalenol’/exp OR ‘alternariol’/exp OR ‘beta zearalenol’/exp OR ‘diacetoxyscirpenol’/exp OR ‘fumonisin’/exp OR ‘fumonisin A1’/exp OR ‘fumonisin B1’/exp OR ‘fumonisin B2’/exp OR ‘fusarenon X’/exp OR ‘HT 2 toxin’/exp OR ‘luteoskyrin’/exp OR ‘masked mycotoxin’/exp OR ‘moniliformin’/exp OR ‘nivalenol’/exp OR ‘ochratoxin’/exp OR ‘patulin’/exp OR ‘sterigmatocystin’/exp OR ‘T 2 toxin’ OR ‘tenuazonic acid’/exp OR ‘trichothecene’/exp OR ‘verrucarin A’/exp OR ‘verrucarin A derivate’/exp OR ‘versicolorin A’/exp OR ‘vomitoxin’/exp OR ‘zearalenone’/exp OR ‘penicillic acid’/exp OR ‘cyclopiazonic acid’/exp OR ‘beauvericin’/exp OR ‘citrinin’/exp OR “enniatin’/exp OR ‘ergot alkaloid’/exp OR ‘fusaric acid’/exp OR ‘neosolaniol’/exp) **AND** (‘central nervous system disease’/exp OR ‘central nervous system*’:ti,ab,kw OR encepalopath*:ti,ab,kw OR hepatoencephalopath*:ti,ab,kw OR neurotox*:ti,ab,kw OR neuropath*:ti,ab,kw OR neurodegenerati*:ti,ab,kw OR ‘parkinson disease’/exp OR parkinson*:ti,ab,kw OR ‘alzheimer disease’/exp OR alzheimer*:ti,ab,kw OR ‘senile dement*’:ti,ab,kw OR neurobehavio*:ti,ab,kw OR ‘nodding syndrome’:ti,ab,kw OR tremor*:ti,ab,kw OR brain:ti,ab,kw OR ‘digestive system disease’/exp OR ‘gastrointestinal disease*’:ti,ab,kw OR ‘gastrointestinal toxic*’:ti,ab,kw OR gastrointestinal*:ti,ab,kw OR intestinal*:ti,ab,kw OR enteropath*:ti,ab,kw OR ‘inflammatory bowel disease’/exp OR ‘inflammatory bowel dis*’:ti,ab,kw OR crohn*:ti,ab,kw OR enteritis:ti,ab,kw OR ileocolitis:ti,ab,kw OR ileitis:ti,ab,kw OR colitis:ti,ab,kw OR ibd:ti,ab,kw OR bowel:ti,ab,kw OR colon:ti,ab,kw OR gut:ti,ab,kw OR ‘kidney disease’/exp OR ‘kidney’/exp OR ‘kidney function test’/exp OR ‘kidney disease*’:ti,ab,kw OR kidney*:ti,ab,kw OR ‘kidney function test*’:ti,ab,kw OR renal:ti,ab,kw OR nephrot*:ti,ab,kw OR anuria:ti,ab,kw OR ‘diabetes insipidus’/exp OR ‘diabetes insipidus’:ti,ab,kw OR nephriti*:ti,ab,kw OR pyelitis:ti,ab,kw OR perinephriti*ti,ab,kw OR glycosuria:ti,ab,kw OR uremia:ti,ab,kw OR polyuri*:ti,ab,kw OR oliguri*:ti,ab,kw OR nephropath*:ti,ab,kw OR ‘liver disease’/exp OR liver*:ti,ab,kw OR hepat*:ti,ab,kw OR cirrhosis*:ti,ab,kw OR ‘esophagus disease’/exp OR ‘esophageal disease*’:ti,ab,kw OR esophagitis*:ti,ab,kw OR ‘hematologic disease’/exp OR ‘lymphatic system disease’/exp OR ‘hematologic disease*’:ti,ab,kw OR anemia:ti,ab,kw OR pancytopenia:ti,ab,kw OR leukopenia:ti,ab,kw OR ‘alimentary toxic aleukia’:ti,ab,kw OR leukocytosis:ti,ab,kw OR ‘lymphatic disease*’:ti,ab,kw OR blood:ti,ab,kw OR ‘lymphatic system’:ti,ab,kw OR ‘immune system’:ti,ab,kw OR ‘immunomodulation’/exp OR immunomodulation:ti,ab,kw OR ‘immunological modulation’:ti,ab,kw OR immunosuppression:ti,ab,kw OR ‘immunological suppression’:ti,ab,kw OR ‘thyroid disease’/exp OR thyroid*:ti,ab,kw OR hyperthyroid*:ti,ab,kw OR hypothyroid*:ti,ab,kw OR goiter*:ti,ab,kw OR graves*:ti,ab,kw OR hyperthyroxinemi*:ti,ab,kw OR hypothyroxinemi*:ti,ab,kw OR thyroxinemi*:ti,ab,kw OR thyrotoxi*:ti,ab,kw OR myxedema*:ti,ab,kw OR myxoedema*:ti,ab,kw OR hashimoto*:ti,ab,kw OR ‘endocrine disease’/exp OR ‘diabetes mellitus’/exp OR ‘endocrine disease*’:ti,ab,kw OR diabetes:ti,ab,kw OR diabetic*:ti,ab,kw OR ‘metabolic syndrome X’/exp OR ‘metabolic syndrome*’:ti,ab,kw OR ‘glucose intolerance’/exp OR ‘glucose intolerance’:ti,ab,kw OR ‘hyperglycemia’/exp OR hyperglycemi*:ti,ab,kw OR ‘insulin resistance’/exp OR ‘insulin resistan*’:ti,ab,kw OR ‘endocrine system’:ti,ab,kw OR ‘cardiovascular disease’/exp OR cardiovascul*:ti,ab,kw OR heart*:ti,ab,kw OR cardiomyo*:ti,ab,kw OR ‘metagenome’/exp OR ‘microflora’/exp OR microbiot*:ti,ab,kw OR microbiom*:ti,ab,kw OR symbios*:ti,ab,kw OR dysbios*:ti,ab,kw OR pathosymbio*:ti,ab,kw OR flora*:ti,ab,kw OR ‘infection’/exp OR infection*ti,ab,kw OR ‘chemically induced disorder’/exp OR ‘environmental disease’/exp OR ‘musculoskeletal disease’/exp OR ‘metabolic disorder’/exp)  Secondary search string:  NOT (‘animal’/exp NOT ‘human’/exp) |
| Reproductive & developmental conditions | Primary search string:  (3-acetyldeoxynivalenol:ti,ab,kw OR 15-acetyldeoxynivalenol:ti,ab,kw OR afla*:ti,ab,kw OR aspertoxin:ti,ab,kw OR ‘aspergillic acid’:ti,ab,kw OR ‘cyclopiazonic acid’:ti,ab,kw OR altertoxin:ti,ab,kw OR alternariol:ti,ab,kw OR ‘alternariol monomethyl ether’:ti,ab,kw OR alpha-zearalanol:ti,ab,kw OR alpha-zearalenol:ti,ab,kw OR altenuene:ti,ab,kw OR beauvericin:ti,ab,kw OR ‘beta-cyclopiazonic acid’:ti,ab,kw OR beta-zearalanol:ti,ab,kw OR beta-zearalenol:ti,ab,kw OR citrinin:ti,ab,kw OR cyclochlorotine:ti,ab,kw OR deoxynivalenol:ti,ab,kw OR vomitoxin:ti,ab,kw OR diacetoxyscirpenol:ti,ab,kw OR dihydro-O-methylsterigmatocystin:ti,ab,kw OR dihydroxy-aflavinine:ti,ab,kw OR dihydroxyl-O-methylsterigmatocystin:ti,ab,kw OR ditryptophenaline:ti,ab,kw OR enniatin*:ti,ab,kw OR ‘ergot alkaloid*’:ti,ab,kw OR flavacol:ti,ab,kw OR fumonisin*:ti,ab,kw OR fusarenon X:ti,ab,kw OR ‘fusaric acid’:ti,ab,kw OR ‘fusarin C’:ti,ab,kw OR ‘HT 2 toxin*’:ti,ab,kw OR ‘HT2 toxin*’:ti,ab,kw OR ‘hydroxyneoaspergillic acid’:ti,ab,kw OR ‘leporin C’:ti,ab,kw OR luteoskyrin:ti,ab,kw OR methylcitreo-isocoumarin:ti,ab,kw OR moniliformin:ti,ab,kw OR mycotoxin*:ti,ab,kw OR neosolaniol:ti,ab,kw OR nivalenol:ti,ab,kw OR ochratoxin*:ti,ab,kw OR O-methylsterigmatocystin:ti,ab,kw OR paspalinine:ti,ab,kw OR pathotoxin:ti,ab,kw OR patulin:ti,ab,kw OR phomopsin:ti,ab,kw OR ‘penicillic acid’:ti,ab,kw OR roquefortine-C:ti,ab,kw OR rugulosin:ti,ab,kw OR ‘speradine A’:ti,ab,kw OR sterigmatocystin:ti,ab,kw OR ‘T2 toxin*’:ti,ab,kw OR ‘T 2 toxin*’:ti,ab,kw OR ‘NX toxin*’:ti,ab,kw OR tentoxin:ti,ab,kw OR ‘tenuazonic acid’:ti,ab,kw OR trichothecene*:ti,ab,kw OR verrucarin:ti,ab,kw OR versiconol:ti,ab,kw OR ‘versiconol hemiacetal acid’:ti,ab,kw OR zearalenone:ti,ab,kw OR zearalanone:ti,ab,kw OR mycotoxin*:ti,ab,kw OR ‘fungal toxin*’:ti,ab,kw OR ‘mycotoxin’/exp OR ‘aflatoxin’/exp OR ‘aflatoxin B’/exp OR ‘aflatoxin B1’/exp OR ‘aflatoxin B2’/exp OR ‘aflatoxin G1’/exp OR ‘aflatoxin G2’/exp OR ‘aflatoxin M1’/exp OR ‘alpha zearalenol’/exp OR ‘alternariol’/exp OR ‘beta zearalenol’/exp OR ‘diacetoxyscirpenol’/exp OR ‘fumonisin’/exp OR ‘fumonisin A1’/exp OR ‘fumonisin B1’/exp OR ‘fumonisin B2’/exp OR ‘fusarenon X’/exp OR ‘HT 2 toxin’/exp OR ‘luteoskyrin’/exp OR ‘masked mycotoxin’/exp OR ‘moniliformin’/exp OR ‘nivalenol’/exp OR ‘ochratoxin’/exp OR ‘patulin’/exp OR ‘sterigmatocystin’/exp OR ‘T 2 toxin’ OR ‘tenuazonic acid’/exp OR ‘trichothecene’/exp OR ‘verrucarin A’/exp OR ‘verrucarin A derivate’/exp OR ‘versicolorin A’/exp OR ‘vomitoxin’/exp OR ‘zearalenone’/exp OR ‘penicillic acid’/exp OR ‘cyclopiazonic acid’/exp OR ‘beauvericin’/exp OR ‘citrinin’/exp OR “enniatin’/exp OR ‘ergot alkaloid’/exp OR ‘fusaric acid’/exp OR ‘neosolaniol’/exp) **AND** (((‘child’/exp OR child*:ti,ab,kw OR newborn*:ti,ab,kw OR neonate*:ti,ab,kw OR neonatal:ti,ab,kw OR ‘infant’/exp OR infant*:ti,ab,kw OR ‘preschool child’/exp OR ‘preschool child*’:ti,ab,kw OR ‘pre school’:ti,ab,kw OR ‘pre-school’:ti,ab,kw OR ‘toddler’/exp OR toddler*:ti,ab,kw OR pediatric*:ti,ab,kw OR paediatric*:ti,ab,kw OR ‘young children*’:ti,ab,kw OR ‘under five years*’:ti,ab,kw OR ‘under 5 years*’:ti,ab,kw OR utero*:ti,ab,kw OR foetal:ti,ab,kw OR fetal:ti,ab,kw) AND (growth*:ti,ab,kw OR stunting*:ti,ab,kw OR stunted*:ti,ab,kw OR wasted*:ti,ab,kw OR wasting*:ti,ab,kw OR underweight*:ti,ab,kw OR ‘short stature*’:ti,ab,kw OR ‘malnutrition’/exp OR malnourished:ti,ab,kw OR ‘mid upper arm circumference’:ti,ab,kw OR ‘mid-upper arm circumference’:ti,ab,kw OR MUAC:ti,ab,kw OR ‘linear growth’:ti,ab,kw OR ‘growth faltering’:ti,ab,kw OR ‘childhood stunting’:ti,ab,kw OR ‘growth impairment’:ti,ab,kw OR ‘growth retardation’:ti,ab,kw OR ‘growth deficit’:ti,ab,kw OR ‘child growth’:ti,ab,kw OR ‘growth restricted’:ti,ab,kw OR birthweight:ti,ab,kw OR ‘birth weight’:ti,ab,kw OR ‘small for gestational age’:ti,ab,kw OR ‘small-for-gestational age’:ti,ab,kw OR length-for-age:ti,ab,kw OR height-for-age*:ti,ab,kw OR weight-for-height:ti,ab,kw OR weight-for-age:ti,ab,kw OR emaciated:ti,ab,kw OR thin:ti,ab,kw OR ‘protein-energy malnutrition’:ti,ab,kw OR ‘immune system’/exp OR ‘immune system’:ti,ab,kw OR ‘immunity’/exp OR ‘immune status’:ti,ab,kw OR antibod*:ti,ab,kw OR enteropath*:ti,ab,kw OR immunosuppression:ti,ab,kw OR immunodeficiency:ti,ab,kw OR immunomodulation:ti,ab,kw OR immunoglobulin*:ti,ab,kw OR immunotoxin*:ti,ab,kw OR immunocompromising:ti,ab,kw OR morbidit*:ti,ab,kw OR ‘infection’/exp OR infection*:ti,ab,kw OR ‘jaundice’/exp OR jaundice:ti,ab,kw OR hepatitis:ti,ab,kw OR outbreak:ti,ab,kw OR marasmus:ti,ab,kw OR kwashiorkor:ti,ab,kw OR ‘marasmic kwashiorkor’:ti,ab,kw OR ‘childhood mortality’/exp OR ‘child mortalit*’:ti,ab,kw OR ‘childhood mortalit*’:ti,ab,kw OR ‘mortality’/exp OR mortalit*:ti,ab,kw OR death OR ‘postnatal mortalit*’:ti,ab,kw OR ‘infant mortality’/exp OR ‘infant mortalit*’:ti,ab,kw OR ‘newborn mortality’/exp OR ‘neonatal mortalit*’:ti,ab,kw OR ‘newborn mortalit*’:ti,ab,kw OR ‘perinatal death’/exp OR ‘perinatal death*’:ti,ab,kw OR ‘postnatal death*’:ti,ab,kw)) OR ‘infertility’/exp OR infertility:ti,ab,kw OR infertile*:ti,ab,kw OR ‘embryo development’/exp OR ‘embryonic development’:ti,ab,kw OR ‘embryo development’:ti,ab,kw OR ‘fetus development’/exp OR ‘fetal development’:ti,ab,kw OR ‘intrauterine growth retardation’/exp OR ‘fetal growth retardation’:ti,ab,kw OR ‘fetus growth’/exp OR ‘fetus growth’:ti,ab,kw OR ‘fetal growth’:ti,ab,kw OR ‘fetal abnormal*’:ti,ab,kw OR ‘placenta’/exp OR placenta*:ti,ab,kw OR ‘pregnancy’/exp OR pregnan*:ti,ab,kw OR mother*:ti,ab,kw OR ‘pregnancy loss’:ti,ab,kw OR ‘spontaneous abortion’:ti,ab,kw OR ‘birth effect’:ti,ab,kw OR ‘adverse pregnancy outcome*’:ti,ab,kw OR ‘pregnancy outcome’/exp OR ‘pregnancy outcome*’:ti,ab,kw OR ‘spontaneous abortion’/exp OR ‘spontaneous abortion’:ti,ab,kw OR abortion:ti,ab,kw OR ‘prematurity’/exp OR ‘premature birth*’:ti,ab,kw OR ‘preterm delivery’:ti,ab,kw OR ‘low birth weight’/exp OR ‘congenital disorder’/exp OR ‘congenital disorder*’:ti,ab,kw OR ‘congenital abnormalit*’:ti,ab,kw OR ‘fetus death’/exp OR ‘fetus death*’:ti,ab,kw OR ‘fetal death*’:ti,ab,kw OR ‘stillbirth’/exp OR stillbirth*:ti,ab,kw OR ‘still birth’:ti,ab,kw)  Secondary search string:  NOT (‘animal’/exp NOT ‘human’/exp) |

**Table S9.** Detailed electronic search string for Cochrane Library.

| **Health risk category** | **Electronic search string – no filters** |
| --- | --- |
| Cancer | Primary search string:  (acetyldeoxynivalenol:ti,ab,kw OR acetyldeoxynivalenol:ti,ab,kw OR afla*:ti,ab,kw OR aspertoxin:ti,ab,kw OR aspergillic NEXT/1 acid:ti,ab,kw OR cyclopiazonic NEXT/1 acid:ti,ab,kw OR altertoxin:ti,ab,kw OR alternariol:ti,ab,kw OR alternariol NEXT/1 monomethyl NEXT/1 ether:ti,ab,kw OR alpha-zearalanol:ti,ab,kw OR alpha-zearalenol:ti,ab,kw OR altenuene:ti,ab,kw OR beauvericin:ti,ab,kw OR beta-cyclopiazonic NEXT/1 acid:ti,ab,kw OR beta-zearalanol:ti,ab,kw OR beta-zearalenol:ti,ab,kw OR citrinin:ti,ab,kw OR cyclochlorotine:ti,ab,kw OR deoxynivalenol:ti,ab,kw OR vomitoxin:ti,ab,kw OR diacetoxyscirpenol:ti,ab,kw OR dihydro-O-methylsterigmatocystin:ti,ab,kw OR dihydroxy-aflavinine:ti,ab,kw OR dihydroxyl-O-methylsterigmatocystin:ti,ab,kw OR ditryptophenaline:ti,ab,kw OR enniatin*:ti,ab,kw OR ergot NEXT/1 alkaloid*:ti,ab,kw OR flavacol:ti,ab,kw OR fumonisin*:ti,ab,kw OR fusarenon:ti,ab,kw OR fusaric NEXT/1 acid:ti,ab,kw OR fusarin NEXT/1 C:ti,ab,kw OR HT NEXT/1 2 NEXT/1 toxin*:ti,ab,kw OR HT2 NEXT/1 toxin*:ti,ab,kw OR hydroxyneoaspergillic NEXT/1 acid:ti,ab,kw OR leporin NEXT/1 C:ti,ab,kw OR luteoskyrin:ti,ab,kw OR methylcitreo-isocoumarin:ti,ab,kw OR moniliformin:ti,ab,kw OR mycotoxin*:ti,ab,kw OR neosolaniol:ti,ab,kw OR nivalenol:ti,ab,kw OR ochratoxin*:ti,ab,kw OR O-methylsterigmatocystin:ti,ab,kw OR paspalinine:ti,ab,kw OR pathotoxin:ti,ab,kw OR patulin:ti,ab,kw OR phomopsin:ti,ab,kw OR penicillic NEXT/1 acid:ti,ab,kw OR roquefortine-C:ti,ab,kw OR rugulosin:ti,ab,kw OR speradine NEXT/1 A:ti,ab,kw OR sterigmatocystin:ti,ab,kw OR T2 NEXT/1 toxin*:ti,ab,kw OR T NEXT/1 2 NEXT/1 toxin*:ti,ab,kw OR NX NEXT/1 toxin*:ti,ab,kw OR tentoxin:ti,ab,kw OR tenuazonic NEXT/1 acid:ti,ab,kw OR trichothecene*:ti,ab,kw OR verrucarin:ti,ab,kw OR versiconol:ti,ab,kw OR versiconol NEXT/1 hemiacetal NEXT/1 acid:ti,ab,kw OR zearalenone:ti,ab,kw OR zearalanone:ti,ab,kw OR mycotoxin*:ti,ab,kw OR fungal NEXT/1 toxin*:ti,ab,kw) **AND** (neoplas*:ti,ab,kw OR paraneoplas*:ti,ab,kw OR tumor*:ti,ab,kw OR tumour*:ti,ab,kw OR cancer*:ti,ab,kw OR carcinogen*:ti,ab,kw OR precancerous:ti,ab,kw OR teratoma*:ti,ab,kw OR malignan*:ti,ab,kw OR oncolog*:ti,ab,kw OR oncogene*:ti,ab,kw OR carcinom*:ti,ab,kw OR sarcom*:ti,ab,kw OR carcinosarcom*:ti,ab,kw OR adenocarcinom*:ti,ab,kw OR adenosarcom*:ti,ab,kw OR adenom*:ti,ab,kw OR melanom*:ti,ab,kw OR gonadoblastom*:ti,ab,kw OR hepatoblastom*:ti,ab,kw OR blastoma*:ti,ab,kw OR chordoma*:ti,ab,kw OR germinoma*:ti,ab,kw OR leukemia*:ti,ab,kw OR lymphoma*:ti,ab,kw OR lymphangioma*:ti,ab,kw OR lymphangiosarcoma*:ti,ab,kw OR meningioma*:ti,ab,kw OR mesenchymoma*:ti,ab,kw OR mesonephroma*:ti,ab,kw OR plasmacytoma*:ti,ab,kw OR teratocarcinoma*:ti,ab,kw OR hodgkin:ti,ab,kw OR non-hodgkin:ti,ab,kw OR myeloma*:ti,ab,kw OR kahler:ti,ab,kw OR metastas*:ti,ab,kw)  Secondary search string:  NOT (animal NOT human) |
| Non-carcinogenic diseases | Primary search string:  (acetyldeoxynivalenol:ti,ab,kw OR acetyldeoxynivalenol:ti,ab,kw OR afla*:ti,ab,kw OR aspertoxin:ti,ab,kw OR aspergillic NEXT/1 acid:ti,ab,kw OR cyclopiazonic NEXT/1 acid:ti,ab,kw OR altertoxin:ti,ab,kw OR alternariol:ti,ab,kw OR alternariol NEXT/1 monomethyl NEXT/1 ether:ti,ab,kw OR alpha-zearalanol:ti,ab,kw OR alpha-zearalenol:ti,ab,kw OR altenuene:ti,ab,kw OR beauvericin:ti,ab,kw OR beta-cyclopiazonic NEXT/1 acid:ti,ab,kw OR beta-zearalanol:ti,ab,kw OR beta-zearalenol:ti,ab,kw OR citrinin:ti,ab,kw OR cyclochlorotine:ti,ab,kw OR deoxynivalenol:ti,ab,kw OR vomitoxin:ti,ab,kw OR diacetoxyscirpenol:ti,ab,kw OR dihydro-O-methylsterigmatocystin:ti,ab,kw OR dihydroxy-aflavinine:ti,ab,kw OR dihydroxyl-O-methylsterigmatocystin:ti,ab,kw OR ditryptophenaline:ti,ab,kw OR enniatin*:ti,ab,kw OR ergot NEXT/1 alkaloid*:ti,ab,kw OR flavacol:ti,ab,kw OR fumonisin*:ti,ab,kw OR fusarenon:ti,ab,kw OR fusaric NEXT/1 acid:ti,ab,kw OR fusarin NEXT/1 C:ti,ab,kw OR HT NEXT/1 2 NEXT/1 toxin*:ti,ab,kw OR HT2 NEXT/1 toxin*:ti,ab,kw OR hydroxyneoaspergillic NEXT/1 acid:ti,ab,kw OR leporin NEXT/1 C:ti,ab,kw OR luteoskyrin:ti,ab,kw OR methylcitreo-isocoumarin:ti,ab,kw OR moniliformin:ti,ab,kw OR mycotoxin*:ti,ab,kw OR neosolaniol:ti,ab,kw OR nivalenol:ti,ab,kw OR ochratoxin*:ti,ab,kw OR O-methylsterigmatocystin:ti,ab,kw OR paspalinine:ti,ab,kw OR pathotoxin:ti,ab,kw OR patulin:ti,ab,kw OR phomopsin:ti,ab,kw OR penicillic NEXT/1 acid:ti,ab,kw OR roquefortine-C:ti,ab,kw OR rugulosin:ti,ab,kw OR speradine NEXT/1 A:ti,ab,kw OR sterigmatocystin:ti,ab,kw OR T2 NEXT/1 toxin*:ti,ab,kw OR T NEXT/1 2 NEXT/1 toxin*:ti,ab,kw OR NX NEXT/1 toxin*:ti,ab,kw OR tentoxin:ti,ab,kw OR tenuazonic NEXT/1 acid:ti,ab,kw OR trichothecene*:ti,ab,kw OR verrucarin:ti,ab,kw OR versiconol:ti,ab,kw OR versiconol NEXT/1 hemiacetal NEXT/1 acid:ti,ab,kw OR zearalenone:ti,ab,kw OR zearalanone:ti,ab,kw OR mycotoxin*:ti,ab,kw OR fungal NEXT/1 toxin*:ti,ab,kw) **AND** (central NEXT/1 nervous NEXT/1 system*:ti,ab,kw OR encepalopath*:ti,ab,kw OR hepatoencephalopath*:ti,ab,kw OR neurotox*:ti,ab,kw OR neuropath*:ti,ab,kw OR neurodegenerati*:ti,ab,kw OR parkinson*:ti,ab,kw OR alzheimer*:ti,ab,kw OR senile NEXT/1 dement*:ti,ab,kw OR neurobehavio*:ti,ab,kw OR nodding NEXT/1 syndrome:ti,ab,kw OR tremor*:ti,ab,kw OR brain:ti,ab,kw OR gastrointestinal NEXT/1 disease*:ti,ab,kw OR gastrointestinal NEXT/1 toxic*:ti,ab,kw OR gastrointestinal*:ti,ab,kw OR intestinal*:ti,ab,kw OR enteropath*:ti,ab,kw OR inflammatory NEXT/1 bowel NEXT/1 dis*:ti,ab,kw OR crohn*:ti,ab,kw OR enteritis:ti,ab,kw OR ileocolitis:ti,ab,kw OR ileitis:ti,ab,kw OR colitis:ti,ab,kw OR ibd:ti,ab,kw OR bowel:ti,ab,kw OR colon:ti,ab,kw OR gut:ti,ab,kw OR kidney NEXT/1 disease*:ti,ab,kw OR kidney*:ti,ab,kw OR kidney NEXT/1 function NEXT/1 test*:ti,ab,kw OR renal:ti,ab,kw OR nephrot*:ti,ab,kw OR anuria:ti,ab,kw OR diabetes NEXT/1 insipidus:ti,ab,kw OR nephriti*:ti,ab,kw OR pyelitis:ti,ab,kw OR perinephriti*ti,ab,kw OR glycosuria:ti,ab,kw OR uremia:ti,ab,kw OR polyuri*:ti,ab,kw OR oliguri*:ti,ab,kw OR nephropath*:ti,ab,kw OR liver*:ti,ab,kw OR hepat*:ti,ab,kw OR cirrhosis*:ti,ab,kw OR esophageal NEXT/1 disease*:ti,ab,kw OR esophagitis*:ti,ab,kw OR hematologic NEXT/1 disease*:ti,ab,kw OR anemia:ti,ab,kw OR pancytopenia:ti,ab,kw OR leukopenia:ti,ab,kw OR alimentary NEXT/1 toxic NEXT/1 aleukia:ti,ab,kw OR leukocytosis:ti,ab,kw OR lymphatic NEXT/1 disease*:ti,ab,kw OR blood:ti,ab,kw OR lymphatic NEXT/1 system:ti,ab,kw OR immune NEXT/1 system:ti,ab,kw OR immunomodulation:ti,ab,kw OR immunological NEXT/1 modulation:ti,ab,kw OR immunosuppression:ti,ab,kw OR immunological NEXT/1 suppression:ti,ab,kw OR thyroid*:ti,ab,kw OR hyperthyroid*:ti,ab,kw OR hypothyroid*:ti,ab,kw OR goiter*:ti,ab,kw OR graves*:ti,ab,kw OR hyperthyroxinemi*:ti,ab,kw OR hypothyroxinemi*:ti,ab,kw OR thyroxinemi*:ti,ab,kw OR thyrotoxi*:ti,ab,kw OR myxedema*:ti,ab,kw OR myxoedema*:ti,ab,kw OR hashimoto*:ti,ab,kw OR endocrine NEXT/1 disease*:ti,ab,kw OR diabetes:ti,ab,kw OR diabetic*:ti,ab,kw OR metabolic NEXT/1 syndrome*:ti,ab,kw OR glucose NEXT/1 intolerance:ti,ab,kw OR hyperglycemi*:ti,ab,kw OR insulin NEXT/1 resistan*:ti,ab,kw OR endocrine NEXT/1 system:ti,ab,kw OR cardiovascul*:ti,ab,kw OR heart*:ti,ab,kw OR cardiomyo*:ti,ab,kw OR microbiot*:ti,ab,kw OR microbiom*:ti,ab,kw OR symbios*:ti,ab,kw OR dysbios*:ti,ab,kw OR pathosymbio*:ti,ab,kw OR flora*:ti,ab,kw OR infection*ti,ab,kw)  Secondary search string:  NOT (animal NOT human) |
| Reproductive & developmental conditions | Primary search string:  (acetyldeoxynivalenol:ti,ab,kw OR acetyldeoxynivalenol:ti,ab,kw OR afla*:ti,ab,kw OR aspertoxin:ti,ab,kw OR aspergillic NEXT/1 acid:ti,ab,kw OR cyclopiazonic NEXT/1 acid:ti,ab,kw OR altertoxin:ti,ab,kw OR alternariol:ti,ab,kw OR alternariol NEXT/1 monomethyl NEXT/1 ether:ti,ab,kw OR alpha-zearalanol:ti,ab,kw OR alpha-zearalenol:ti,ab,kw OR altenuene:ti,ab,kw OR beauvericin:ti,ab,kw OR beta-cyclopiazonic NEXT/1 acid:ti,ab,kw OR beta-zearalanol:ti,ab,kw OR beta-zearalenol:ti,ab,kw OR citrinin:ti,ab,kw OR cyclochlorotine:ti,ab,kw OR deoxynivalenol:ti,ab,kw OR vomitoxin:ti,ab,kw OR diacetoxyscirpenol:ti,ab,kw OR dihydro-O-methylsterigmatocystin:ti,ab,kw OR dihydroxy-aflavinine:ti,ab,kw OR dihydroxyl-O-methylsterigmatocystin:ti,ab,kw OR ditryptophenaline:ti,ab,kw OR enniatin*:ti,ab,kw OR ergot NEXT/1 alkaloid*:ti,ab,kw OR flavacol:ti,ab,kw OR fumonisin*:ti,ab,kw OR fusarenon:ti,ab,kw OR fusaric NEXT/1 acid:ti,ab,kw OR fusarin NEXT/1 C:ti,ab,kw OR HT NEXT/1 2 NEXT/1 toxin*:ti,ab,kw OR HT2 NEXT/1 toxin*:ti,ab,kw OR hydroxyneoaspergillic NEXT/1 acid:ti,ab,kw OR leporin NEXT/1 C:ti,ab,kw OR luteoskyrin:ti,ab,kw OR methylcitreo-isocoumarin:ti,ab,kw OR moniliformin:ti,ab,kw OR mycotoxin*:ti,ab,kw OR neosolaniol:ti,ab,kw OR nivalenol:ti,ab,kw OR ochratoxin*:ti,ab,kw OR O-methylsterigmatocystin:ti,ab,kw OR paspalinine:ti,ab,kw OR pathotoxin:ti,ab,kw OR patulin:ti,ab,kw OR phomopsin:ti,ab,kw OR penicillic NEXT/1 acid:ti,ab,kw OR roquefortine-C:ti,ab,kw OR rugulosin:ti,ab,kw OR speradine NEXT/1 A:ti,ab,kw OR sterigmatocystin:ti,ab,kw OR T2 NEXT/1 toxin*:ti,ab,kw OR T NEXT/1 2 NEXT/1 toxin*:ti,ab,kw OR NX NEXT/1 toxin*:ti,ab,kw OR tentoxin:ti,ab,kw OR tenuazonic NEXT/1 acid:ti,ab,kw OR trichothecene*:ti,ab,kw OR verrucarin:ti,ab,kw OR versiconol:ti,ab,kw OR versiconol NEXT/1 hemiacetal NEXT/1 acid:ti,ab,kw OR zearalenone:ti,ab,kw OR zearalanone:ti,ab,kw OR mycotoxin*:ti,ab,kw OR fungal NEXT/1 toxin*:ti,ab,kw) **AND** (((child*:ti,ab,kw OR newborn*:ti,ab,kw OR neonate*:ti,ab,kw OR neonatal:ti,ab,kw OR infant*:ti,ab,kw OR preschool NEXT/1 child*:ti,ab,kw OR pre NEXT/1 school:ti,ab,kw OR pre-school:ti,ab,kw OR toddler*:ti,ab,kw OR pediatric*:ti,ab,kw OR paediatric*:ti,ab,kw OR young NEXT/1 children*:ti,ab,kw OR under NEXT/1 five NEXT/1 years*:ti,ab,kw OR under NEXT/1 5 NEXT/1 years*:ti,ab,kw OR utero*:ti,ab,kw OR foetal:ti,ab,kw OR fetal:ti,ab,kw) AND (growth*:ti,ab,kw OR stunting*:ti,ab,kw OR stunted*:ti,ab,kw OR wasted*:ti,ab,kw OR wasting*:ti,ab,kw OR underweight*:ti,ab,kw OR short NEXT/1 stature*:ti,ab,kw OR malnourished:ti,ab,kw OR mid NEXT/1 upper NEXT/1 arm NEXT/1 circumference:ti,ab,kw OR mid-upper NEXT/1 arm NEXT/1 circumference:ti,ab,kw OR MUAC:ti,ab,kw OR linear NEXT/1 growth:ti,ab,kw OR growth NEXT/1 faltering:ti,ab,kw OR childhood NEXT/1 stunting:ti,ab,kw OR growth NEXT/1 impairment:ti,ab,kw OR growth NEXT/1 retardation:ti,ab,kw OR growth NEXT/1 deficit:ti,ab,kw OR child NEXT/1 growth:ti,ab,kw OR growth NEXT/1 restricted:ti,ab,kw OR birthweight:ti,ab,kw OR birth NEXT/1 weight:ti,ab,kw OR small NEXT/1 for NEXT/1 gestational NEXT/1 age:ti,ab,kw OR small-for-gestational NEXT/1 age:ti,ab,kw OR length-for-age:ti,ab,kw OR height-for-age*:ti,ab,kw OR weight-for-height:ti,ab,kw OR weight-for-age:ti,ab,kw OR emaciated:ti,ab,kw OR thin:ti,ab,kw OR protein-energy NEXT/1 malnutrition:ti,ab,kw OR immune NEXT/1 system:ti,ab,kw OR immune NEXT/1 status:ti,ab,kw OR antibod*:ti,ab,kw OR enteropath*:ti,ab,kw OR immunosuppression:ti,ab,kw OR immunodeficiency:ti,ab,kw OR immunomodulation:ti,ab,kw OR immunoglobulin*:ti,ab,kw OR immunotoxin*:ti,ab,kw OR immunocompromising:ti,ab,kw OR morbidit*:ti,ab,kw OR infection*:ti,ab,kw OR jaundice:ti,ab,kw OR hepatitis:ti,ab,kw OR outbreak:ti,ab,kw OR marasmus:ti,ab,kw OR kwashiorkor:ti,ab,kw OR marasmic NEXT/1 kwashiorkor:ti,ab,kw OR child NEXT/1 mortalit*:ti,ab,kw OR childhood NEXT/1 mortalit*:ti,ab,kw OR mortalit*:ti,ab,kw OR death OR postnatal NEXT/1 mortalit*:ti,ab,kw OR infant NEXT/1 mortalit*:ti,ab,kw OR neonatal NEXT/1 mortalit*:ti,ab,kw OR newborn NEXT/1 mortalit*:ti,ab,kw OR perinatal NEXT/1 death*:ti,ab,kw OR postnatal NEXT/1 death*:ti,ab,kw)) OR infertility:ti,ab,kw OR infertile*:ti,ab,kw OR embryonic NEXT/1 development:ti,ab,kw OR embryo NEXT/1 development:ti,ab,kw OR fetal NEXT/1 development:ti,ab,kw OR fetal NEXT/1 growth NEXT/1 retardation:ti,ab,kw OR fetus NEXT/1 growth:ti,ab,kw OR fetal NEXT/1 growth:ti,ab,kw OR fetal NEXT/1 abnormal*:ti,ab,kw OR placenta*:ti,ab,kw OR pregnan*:ti,ab,kw OR mother*:ti,ab,kw OR pregnancy NEXT/1 loss:ti,ab,kw OR spontaneous NEXT/1 abortion:ti,ab,kw OR birth NEXT/1 effect:ti,ab,kw OR adverse NEXT/1 pregnancy NEXT/1 outcome*:ti,ab,kw OR pregnancy NEXT/1 outcome*:ti,ab,kw OR spontaneous NEXT/1 abortion:ti,ab,kw OR abortion:ti,ab,kw OR premature NEXT/1 birth*:ti,ab,kw OR preterm NEXT/1 delivery:ti,ab,kw OR congenital NEXT/1 disorder*:ti,ab,kw OR congenital NEXT/1 abnormalit*:ti,ab,kw OR fetus NEXT/1 death*:ti,ab,kw OR fetal NEXT/1 death*:ti,ab,kw OR stillbirth*:ti,ab,kw OR still NEXT/1 birth:ti,ab,kw)  Secondary search string:  NOT (animal NOT human) |

**Table S10.** Detailed electronic search string for Web of Science.

| **Health risk category** | **Electronic search string – no filters** |
| --- | --- |
| Cancer | Primary search string:  (3-acetyldeoxynivalenol OR 15-acetyldeoxynivalenol OR afla* OR aspertoxin OR “aspergillic acid” OR “cyclopiazonic acid” OR altertoxin OR alternariol OR “alternariol monomethyl ether” OR alpha-zearalanol OR alpha-zearalenol OR altenuene OR beauvericin OR beta-cyclopiazonic acid OR beta-zearalanol OR beta-zearalenol OR citrinin OR cyclochlorotine OR deoxynivalenol OR vomitoxin OR diacetoxyscirpenol OR dihydro-O-methylsterigmatocystin OR dihydroxy-aflavinine OR dihydroxyl-O-methylsterigmatocystin OR ditryptophenaline OR enniatin* OR “ergot alkaloid*” OR flavacol OR fumonisin* OR “fusarenon X” OR “fusaric acid” OR “fusarin C” OR “HT2 toxin” OR “HT 2 toxin” OR “hydroxyneoaspergillic acid” OR “leporin C” OR luteoskyrin OR methylcitreo-isocoumarin OR moniliformin OR mycotoxin* OR neosolaniol OR nivalenol OR ochratoxin* OR O-methylsterigmatocystin OR paspalinine OR pathotoxin OR patulin OR phomopsin OR “penicillic acid” OR “roquefortine C” OR rugulosin OR speradine A OR sterigmatocystin OR “T2 toxin” OR “T 2 toxin” OR “NX toxin” OR tentoxin OR “tenuazonic acid” OR trichothecene* OR verrucarin OR versiconol OR zearalenone OR zearalanol OR zearalenol OR mycotoxin* OR “fungal toxin*”) **AND** (neoplas* OR paraneoplas* OR tumor* OR tumour* OR cancer* OR carcinogen* OR precancerous OR teratoma* OR malignan* OR oncolog* OR oncogene* OR carcinom* OR sarcom* OR carcinosarcom* OR adenocarcinom* OR adenosarcom* OR adenom* OR melanom* OR gonadoblastom* OR hepatoblastom* OR blastoma* OR chordoma* OR germinoma* OR leukemia* OR lymphoma* OR lymphangioma* OR lymphangiosarcoma* OR meningioma* OR mesenchymoma* OR mesonephroma* OR plasmacytoma* OR teratocarcinoma* OR hodgkin OR non-hodgkin OR myeloma* OR kahler OR metastas*)  Secondary search string:  NOT (Animal NOT Human) |
| Non-carcinogenic diseases | Primary search string:  (3-acetyldeoxynivalenol OR 15-acetyldeoxynivalenol OR afla* OR aspertoxin OR “aspergillic acid” OR “cyclopiazonic acid” OR altertoxin OR alternariol OR “alternariol monomethyl ether” OR alpha-zearalanol OR alpha-zearalenol OR altenuene OR beauvericin OR beta-cyclopiazonic acid OR beta-zearalanol OR beta-zearalenol OR citrinin OR cyclochlorotine OR deoxynivalenol OR vomitoxin OR diacetoxyscirpenol OR dihydro-O-methylsterigmatocystin OR dihydroxy-aflavinine OR dihydroxyl-O-methylsterigmatocystin OR ditryptophenaline OR enniatin* OR “ergot alkaloid*” OR flavacol OR fumonisin* OR “fusarenon X” OR “fusaric acid” OR “fusarin C” OR “HT2 toxin” OR “HT 2 toxin” OR “hydroxyneoaspergillic acid” OR “leporin C” OR luteoskyrin OR methylcitreo-isocoumarin OR moniliformin OR mycotoxin* OR neosolaniol OR nivalenol OR ochratoxin* OR O-methylsterigmatocystin OR paspalinine OR pathotoxin OR patulin OR phomopsin OR “penicillic acid” OR “roquefortine C” OR rugulosin OR speradine A OR sterigmatocystin OR “T2 toxin” OR “T 2 toxin” OR “NX toxin” OR tentoxin OR “tenuazonic acid” OR trichothecene* OR verrucarin OR versiconol OR zearalenone OR zearalanol OR zearalenol OR mycotoxin* OR “fungal toxin*”) **AND**  (“central nervous system disease*” OR encephalopath* OR hepatoencephalopath* OR neurotoxi* OR neuropath* OR neurodegenerati* OR parkinson* OR alzheimer* OR “senile dementia*” OR neurobehavio* OR “nodding syndrome” OR tremor* OR “brain” OR “gastrointestinal disease*” OR “gastrointestinal toxic*” OR enteropath* OR “inflammatory bowel dis*” OR crohn* OR ileocolitis OR ileitis OR ibd OR colitis OR enteritis OR gastrointestin* OR intestine* OR bowel OR colon OR nephrot* OR anuria OR “diabetes insipidus” OR nephriti* OR pyelitis OR perinephriti* OR glycosuria OR uremia OR polyuri* OR oliguri* OR nephropath* OR kidney OR renal OR hepat* OR cirrhosis OR liver* OR “esophageal disease*” OR esophagitis OR esophagus OR “hematologic disease*” OR anemia OR pancytopenia OR leukopenia OR “alimentary toxic aleukia” OR leukocytosis OR “lymphatic disease*” OR blood OR “lymphatic system” OR “immune system” OR immunomodulation OR “immunological modulation” OR immunosuppression OR “immunological suppression” OR thyroid OR hyperthyroid* OR hypothyroid* OR goiter* OR graves* OR hyperthyroxinemi* OR hypothyroxinemi* OR thyroxinemi* OR Thyrotoxi* OR myxedema* OR myxoedema* OR hashimoto* OR thyroid OR diabetes* OR diabetic* OR “metabolic syndrome” OR “glucose intoleran*” OR hyperglycemi* OR “insulin resistan*” OR “endocrine system” OR cardiovascul* OR heart OR cardiomyo* OR metagenom* OR microbiom* OR microbiot* OR symbios* OR dysbios* OR pathosymbio* OR flora* OR microflora* OR infection*)  Secondary search string:  NOT (Animal NOT Human) |
| Reproductive & developmental conditions | Primary search string:  (3-acetyldeoxynivalenol OR 15-acetyldeoxynivalenol OR afla* OR aspertoxin OR “aspergillic acid” OR “cyclopiazonic acid” OR altertoxin OR alternariol OR “alternariol monomethyl ether” OR alpha-zearalanol OR alpha-zearalenol OR altenuene OR beauvericin OR beta-cyclopiazonic acid OR beta-zearalanol OR beta-zearalenol OR citrinin OR cyclochlorotine OR deoxynivalenol OR vomitoxin OR diacetoxyscirpenol OR dihydro-O-methylsterigmatocystin OR dihydroxy-aflavinine OR dihydroxyl-O-methylsterigmatocystin OR ditryptophenaline OR enniatin* OR “ergot alkaloid*” OR flavacol OR fumonisin* OR “fusarenon X” OR “fusaric acid” OR “fusarin C” OR “HT2 toxin” OR “HT 2 toxin” OR “hydroxyneoaspergillic acid” OR “leporin C” OR luteoskyrin OR methylcitreo-isocoumarin OR moniliformin OR mycotoxin* OR neosolaniol OR nivalenol OR ochratoxin* OR O-methylsterigmatocystin OR paspalinine OR pathotoxin OR patulin OR phomopsin OR “penicillic acid” OR “roquefortine C” OR rugulosin OR speradine A OR sterigmatocystin OR “T2 toxin” OR “T 2 toxin” OR “NX toxin” OR tentoxin OR “tenuazonic acid” OR trichothecene* OR verrucarin OR versiconol OR zearalenone OR zearalanol OR zearalenol OR mycotoxin* OR “fungal toxin*”)  **AND** (((Child* OR Newborn* OR neonate* OR neonatal OR infant* OR preschool OR “pre school” OR pre-school OR toddler* OR pediatric* OR paediatric OR “young children” OR “under five years” OR “under 5 years” OR utero OR foetal OR fetal) AND (growth OR stunting OR stunted OR wasted OR wasting OR underweight OR “short stature” OR malnutrition OR malnourished OR “mid upper arm circumference” OR “mid-upper arm circumference” OR MUAC OR “linear growth” OR “growth faltering” OR “childhood stunting” OR “growth impairment” OR “growth retardation” OR “growth deficit” OR ”child growth” OR “growth restricted” OR birthweight OR “birth weight” OR “small for gestational age” OR “small-for-gestational age” OR length-for-age OR height-for-age OR weight-for-height OR weight-for-age OR emaciated OR thin OR “protein-energy malnutrition” OR “immune system” OR “immune status” OR antibody OR enteropathy OR immunosuppression OR immunodeficiency OR immunomodulation OR immunoglobulin OR immunotoxin OR immunocompromising OR morbidity OR infection* OR jaundice OR hepatitis OR outbreak OR marasmus OR kwashiorkor OR “marasmic kwashiorkor” OR mortality OR death OR “postnatal mortality” OR “neonatal mortality” OR “postnatal death”)) OR infertile* OR “embryo* development” OR “fetal development*” OR “fetal growth*” OR “fetal abnormal*” OR placenta* OR pregnan* OR mother* OR maternal OR “adverse pregnancy outcome*” OR miscarriage OR “pregnancy loss” OR abortion* OR “premature birth” OR “preterm delivery” OR “congenital abnormalit*” OR fetal death OR stillbirth OR “still birth” OR “pregnancy outcome*”)  Secondary search string:  NOT (Animal NOT Human) |

**Table S11.** Detailed electronic search string for Scopus.

| **Health risk category** | **Electronic search string – no filters** |
| --- | --- |
| Cancer | Primary search string:  TITLE-ABS-KEY ((3-acetyldeoxynivalenol OR 15-acetyldeoxynivalenol OR afla* OR aspertoxin OR “aspergillic acid” OR “cyclopiazonic acid” OR altertoxin OR alternariol OR “alternariol monomethyl ether” OR alpha-zearalanol OR alpha-zearalenol OR altenuene OR beauvericin OR beta-cyclopiazonic acid OR beta-zearalanol OR beta-zearalenol OR citrinin OR cyclochlorotine OR deoxynivalenol OR vomitoxin OR diacetoxyscirpenol OR dihydro-O-methylsterigmatocystin OR dihydroxy-aflavinine OR dihydroxyl-O-methylsterigmatocystin OR ditryptophenaline OR enniatin* OR “ergot alkaloid*” OR flavacol OR fumonisin* OR “fusarenon X” OR “fusaric acid” OR “fusarin C” OR “HT2 toxin” OR “HT 2 toxin” OR “hydroxyneoaspergillic acid” OR “leporin C” OR luteoskyrin OR methylcitreo-isocoumarin OR moniliformin OR mycotoxin* OR neosolaniol OR nivalenol OR ochratoxin* OR O-methylsterigmatocystin OR paspalinine OR pathotoxin OR patulin OR phomopsin OR “penicillic acid” OR “roquefortine C” OR rugulosin OR speradine A OR sterigmatocystin OR “T2 toxin” OR “T 2 toxin” OR “NX toxin” OR tentoxin OR “tenuazonic acid” OR trichothecene* OR verrucarin OR versiconol OR zearalenone OR zearalanol OR zearalenol OR mycotoxin* OR “fungal toxin*”) **AND** (neoplas* OR paraneoplas* OR tumor* OR tumour* OR cancer* OR carcinogen* OR precancerous OR teratoma* OR malignan* OR oncolog* OR oncogene* OR carcinom* OR sarcom* OR carcinosarcom* OR adenocarcinom* OR adenosarcom* OR adenom* OR melanom* OR gonadoblastom* OR hepatoblastom* OR blastoma* OR chordoma* OR germinoma* OR leukemia* OR lymphoma* OR lymphangioma* OR lymphangiosarcoma* OR meningioma* OR mesenchymoma* OR mesonephroma* OR plasmacytoma* OR teratocarcinoma* OR hodgkin OR non-hodgkin OR myeloma* OR kahler OR metastas*))  Secundary search string:  TITLE-ABS-KEY ((#1 AND #2) NOT (Animal not Human)) |
| Non-carcinogenic diseases | Primary search string:  TITLE-ABS-KEY ((3-acetyldeoxynivalenol OR 15-acetyldeoxynivalenol OR afla* OR aspertoxin OR “aspergillic acid” OR “cyclopiazonic acid” OR altertoxin OR alternariol OR “alternariol monomethyl ether” OR alpha-zearalanol OR alpha-zearalenol OR altenuene OR beauvericin OR beta-cyclopiazonic acid OR beta-zearalanol OR beta-zearalenol OR citrinin OR cyclochlorotine OR deoxynivalenol OR vomitoxin OR diacetoxyscirpenol OR dihydro-O-methylsterigmatocystin OR dihydroxy-aflavinine OR dihydroxyl-O-methylsterigmatocystin OR ditryptophenaline OR enniatin* OR “ergot alkaloid*” OR flavacol OR fumonisin* OR “fusarenon X” OR “fusaric acid” OR “fusarin C” OR “HT2 toxin” OR “HT 2 toxin” OR “hydroxyneoaspergillic acid” OR “leporin C” OR luteoskyrin OR methylcitreo-isocoumarin OR moniliformin OR mycotoxin* OR neosolaniol OR nivalenol OR ochratoxin* OR O-methylsterigmatocystin OR paspalinine OR pathotoxin OR patulin OR phomopsin OR “penicillic acid” OR “roquefortine C” OR rugulosin OR speradine A OR sterigmatocystin OR “T2 toxin” OR “T 2 toxin” OR “NX toxin” OR tentoxin OR “tenuazonic acid” OR trichothecene* OR verrucarin OR versiconol OR zearalenone OR zearalanol OR zearalenol OR mycotoxin* OR “fungal toxin*”) **AND** (“central nervous system disease*” OR encephalopath* OR hepatoencephalopath* OR neurotoxi* OR neuropath* OR neurodegenerati* OR parkinson* OR alzheimer* OR “senile dementia*” OR neurobehavio* OR “nodding syndrome” OR tremor* OR “brain” OR “gastrointestinal disease*” OR “gastrointestinal toxic*” OR enteropath* OR “inflammatory bowel dis*” OR crohn* OR ileocolitis OR ileitis OR ibd OR colitis OR enteritis OR gastrointestin* OR intestine* OR bowel OR colon OR nephrot* OR anuria OR “diabetes insipidus” OR nephriti* OR pyelitis OR perinephriti* OR glycosuria OR uremia OR polyuri* OR oliguri* OR nephropath* OR kidney OR renal OR hepat* OR cirrhosis OR liver* OR “esophageal disease*” OR esophagitis OR esophagus OR “hematologic disease*” OR anemia OR pancytopenia OR leukopenia OR “alimentary toxic aleukia” OR leukocytosis OR “lymphatic disease*” OR blood OR “lymphatic system” OR “immune system” OR immunomodulation OR “immunological modulation” OR immunosuppression OR “immunological suppression” OR thyroid OR hyperthyroid* OR hypothyroid* OR goiter* OR graves* OR hyperthyroxinemi* OR hypothyroxinemi* OR thyroxinemi* OR Thyrotoxi* OR myxedema* OR myxoedema* OR hashimoto* OR thyroid OR diabetes* OR diabetic* OR “metabolic syndrome” OR “glucose intoleran*” OR hyperglycemi* OR “insulin resistan*” OR “endocrine system” OR cardiovascul* OR heart OR cardiomyo* OR metagenom* OR microbiom* OR microbiot* OR symbios* OR dysbios* OR pathosymbio* OR flora* OR microflora* OR infection*))  Secundary search string:  TITLE-ABS-KEY ((#1 AND #2) NOT (Animal not Human)) |
| Reproductive & developmental conditions | Primary search string:  TITLE-ABS-KEY ((3-acetyldeoxynivalenol OR 15-acetyldeoxynivalenol OR afla* OR aspertoxin OR “aspergillic acid” OR “cyclopiazonic acid” OR altertoxin OR alternariol OR “alternariol monomethyl ether” OR alpha-zearalanol OR alpha-zearalenol OR altenuene OR beauvericin OR beta-cyclopiazonic acid OR beta-zearalanol OR beta-zearalenol OR citrinin OR cyclochlorotine OR deoxynivalenol OR vomitoxin OR diacetoxyscirpenol OR dihydro-O-methylsterigmatocystin OR dihydroxy-aflavinine OR dihydroxyl-O-methylsterigmatocystin OR ditryptophenaline OR enniatin* OR “ergot alkaloid*” OR flavacol OR fumonisin* OR “fusarenon X” OR “fusaric acid” OR “fusarin C” OR “HT2 toxin” OR “HT 2 toxin” OR “hydroxyneoaspergillic acid” OR “leporin C” OR luteoskyrin OR methylcitreo-isocoumarin OR moniliformin OR mycotoxin* OR neosolaniol OR nivalenol OR ochratoxin* OR O-methylsterigmatocystin OR paspalinine OR pathotoxin OR patulin OR phomopsin OR “penicillic acid” OR “roquefortine C” OR rugulosin OR speradine A OR sterigmatocystin OR “T2 toxin” OR “T 2 toxin” OR “NX toxin” OR tentoxin OR “tenuazonic acid” OR trichothecene* OR verrucarin OR versiconol OR zearalenone OR zearalanol OR zearalenol OR mycotoxin* OR “fungal toxin*”) **AND** (((Child* OR Newborn* OR neonate* OR neonatal OR infant* OR preschool OR “pre school” OR pre-school OR toddler* OR pediatric* OR paediatric OR “young children” OR “under five years” OR “under 5 years” OR utero OR foetal OR fetal) AND (growth OR stunting OR stunted OR wasted OR wasting OR underweight OR “short stature” OR malnutrition OR malnourished OR “mid upper arm circumference” OR “mid-upper arm circumference” OR MUAC OR “linear growth” OR “growth faltering” OR “childhood stunting” OR “growth impairment” OR “growth retardation” OR “growth deficit” OR ”child growth” OR “growth restricted” OR birthweight OR “birth weight” OR “small for gestational age” OR “small-for-gestational age” OR length-for-age OR height-for-age OR weight-for-height OR weight-for-age OR emaciated OR thin OR “protein-energy malnutrition” OR “immune system” OR “immune status” OR antibody OR enteropathy OR immunosuppression OR immunodeficiency OR immunomodulation OR immunoglobulin OR immunotoxin OR immunocompromising OR morbidity OR infection* OR jaundice OR hepatitis OR outbreak OR marasmus OR kwashiorkor OR “marasmic kwashiorkor” OR mortality OR death OR “postnatal mortality” OR “neonatal mortality” OR “postnatal death”)) OR infertile* OR “embryo* development” OR “fetal development*” OR “fetal growth*” OR “fetal abnormal*” OR placenta* OR pregnan* OR mother* OR maternal OR “adverse pregnancy outcome*” OR miscarriage OR “pregnancy loss” OR abortion* OR “premature birth” OR “preterm delivery” OR “congenital abnormalit*” OR fetal death OR stillbirth OR “still birth” OR “pregnancy outcome*”))  Secundary search string:  TITLE-ABS-KEY ((#1 AND #2) NOT (Animal not Human)) |

**Table S12.** An example of the tabular format covering the characteristics of the included studies and the outcomes of interest for inclusion into the systematic review report.

| **N°** | **Study design** | **Population** | **Study period** | **Study duration** | **M/F** | **N** | **Mean age**  **(± SD)** | **Cases** | **Controls** | **Method**  **LOD;LOQ** | **Matrix/type of food** | **Detection rate (%)** | **Adjustment for covariates** | **OR, RR, SMD, IRR, CC** | **Other outcomes** |
| --- | --- | --- | --- | --- | --- | --- | --- | --- | --- | --- | --- | --- | --- | --- | --- |
| Aflatoxins – Liver disease | | | | | | | | | | | | | | | |
| Farag et al. 2018^1^ | Case-control | Saudi Arabia | 01/2013-06/2014 | 1.5 year | - | 50 | - | 38 | 12 | HPLC-FLD + ELISA  LOQ = 0.5 µg/L | Serum | 17% | Age and dietary intake | CC = 0.687* CC = 0.503** | - |

Note: M/F = male/female ratio, N = number of participants, LOD = limit of detection, LOQ = limit of quantification, OR = odds ratio, RR = risk ratio, SMD = standardized mean difference, IRR = incidence rate ratio, CC = correlation coefficient, - = not reported, AFB1 = aflatoxin B1, HPLC-FLD = high performance liquid chromatography coupled to fluorescence detection, ELISA = enzyme-linked immunosorbent assay, * = elevated liver enzyme ALT, ** = elevated liver enzyme AST.

**
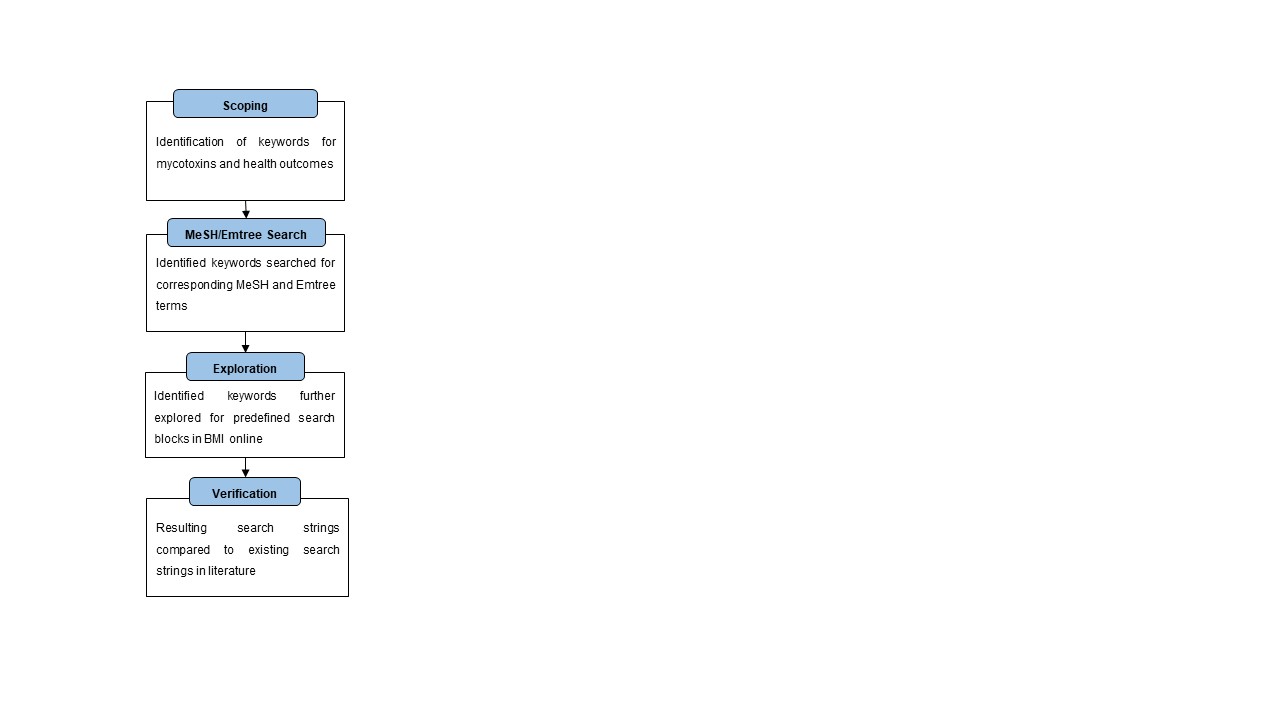
**

**Figure S1.** Schematic overview of the search string development. MeSH = Medical Subject Headings, BMI = BioMedical Information ^2^.

**References**

1. Farag, R. M. M. A., AlAyobi, D., Kwon, H. J. & EL-Ansary, A. Relationship between aflatoxin B1 exposure and etiology of liver disease in Saudi Arabian patients. *J. Pure Appl. Microbiol.* **12**, 1147–1153 (2018).

2. BioMedical information. BMI Search Blocks. https://blocks.bmi-online.nl/ (2020).
